# Firing discrimination: Selective labor market responses of firms during the COVID-19 economic crisis

**DOI:** 10.1371/journal.pone.0262337

**Published:** 2022-01-31

**Authors:** Daniel Auer

**Affiliations:** University of Mannheim, MZES, Mannheim, Germany; Ghent University: Universiteit Gent, BELGIUM

## Abstract

The speed of the economic downturn in the wake of the COVID-19 pandemic has been exceptional, causing mass layoffs—in Germany up to 30% of the workforce in some industries. Economic rationale suggests that the decision on which workers are fired should depend on productivity-related individual factors. However, from hiring situations we know that discrimination—i.e., decisions driven by characteristics unrelated to productivity—is widespread in Western labor markets. Drawing on representative survey data on forced layoffs and short-time work collected in Germany between April and December 2020, this study highlights that discrimination against immigrants is also present in firing situations. The analysis shows that employees with a migration background are significantly more likely to lose their job than native workers when otherwise healthy firms are unexpectedly forced to let go of part of their workforce, while firms make more efforts to substitute firing with short-time working schemes for their native workers. Adjusting for detailed job-related characteristics shows that the findings are unlikely to be driven by systematic differences in productivity between migrants and natives. Moreover, using industry-specific variation in the extent of the economic downturn, I demonstrate that layoff probabilities hardly differ across the less affected industries, but that the gap between migrants and natives increases with the magnitude of the shock. In the hardest-hit industries, job loss probability among migrants is three times higher than among natives. This confirms the hypothesis that firing discrimination puts additional pressure on the immigrant workforce in times of crisis.

## 1. Introduction

The COVID-19 pandemic starting in early 2020 has not only caused a massive health crisis but also an economic shock that has been exceptional in terms of the magnitude and speed of the downturn. Almost every country was forced to drastically limit public life and to shut down—at least temporarily—its service, tourism, education, and even parts of the manufacturing industries. This abrupt slowdown of the global economy has led to unprecedented mass layoffs, despite recent studies suggesting an overall successful introduction of targeted policies to mitigate the impact on employment, such as short-time work [1]. In the U.S. estimates suggest that 20 million people lost their jobs by April 2020, substantially exceeding the impact of the 1930s’ Great Depression [2]. In some industries of the German economy, up to 30% of the workforce has been dismissed [3], while direct measures, such as closures of non-essential businesses, only explain a small fraction of the rise in unemployment [4]. This suggests that most of the negative short-run shock was due to a decline in consumer demand, stay-at-home orders, and general containment measures, which affected the economy across (almost) all industries and independent of past firm performance.

However, the burden of the crisis is not equally distributed across the affected societies. In the wake of the COVID-19 pandemic, the employment rate of Black and Hispanic workers in the U.S. declined disproportionally between April and May 2020 [5] and occupational sorting only partly explains this gap [6, 7]. Similarly, determinants of higher socioeconomic status in China are associated with less income loss [8], thus exacerbating pre-existing inequalities. Moehring et al. [9] link the inequality in job loss and furlough patterns in Germany to educational attainment and—related to that—to the possibility of working from home. They find that the pandemic disproportionately affected low-skilled workers.

In this study, I use the major economic downturn that accompanies the COVID-19 pandemic as an exogenous shock to provide evidence on substantial *firing discrimination* against migrant workers. If not stated otherwise, I also refer to people with a migration background (at least one parent born abroad; the official definition) as migrants. In doing so, I link the patterns of growing inequality in the wake of a crisis to discriminatory behavior on behalf of employers. Moreover, I go beyond existing studies on discrimination that typically focus on the *hiring* phase. While research on hiring discrimination faces the difficulty to disentangle firms’ animus toward minorities from behavior in the face of uncertainty about the actual productivity of job applicants [10 vs. 11, 12], the firing of proven workers is arguably less driven by statistical discrimination but by stereotypes (taste or animus [10]). That is, hired workers have already proven their productivity, so that differences in layoff propensity are significantly less likely affected by unobserved productivity differentials [13]. I thus contribute to the ongoing debate on the mechanisms of ethnic labor market discrimination (taste-based vs. statistical discrimination) and corroborate a small body of literature stressing that discrimination continues throughout the working life and does not stop after the hiring stage [14–16].

Previous studies have found increasing inequalities during the economic downturn [17]. However, they do not cleanly separate firm-level behavior from individual productivity-related characteristics and sorting effects. A major challenge for identifying systematic differences in job loss between migrants and natives—i.e., discrimination—is the sorting of migrants into industries, occupations, and firms [18–21]. Borjas and Cassidy [22], for instance, argue that the relative increase in the immigrant rate of job loss during the pandemic is due to this sorting, as migrants are more likely to have occupations with limited teleworking possibilities and less likely to hold “remotable” skills. Paggiaro [23] and Bratsberg et al. [24] similarly argue that the sorting of immigrants into companies and industries with a higher vulnerability to economic shocks is responsible for the observed widening of inequality during a recession or at least blurs discriminatory behavior on behalf of employers.

To account for heterogeneous selection of migrants and natives into industries and occupations, I leverage representative survey data from Germany collected (bi-)weekly between April and December 2020 (N = 11,440) and combine it with detailed official statistics on unemployment trends at the industry level. According to this data, large shares of both migrants and natives who have been employed prior to the onset of the pandemic experienced employment cuts and firing. 24% of the migrants and 20% of the natives in the sample report take-up of short-time work. Short-time work is a crisis measure (lastly in effect on a large scale during the economic crisis in 2007 onward) introduced—and subsidized—by the German government to enable firms to keep their workers on reduced hours and pay instead of firing them. At the same time, among pre-pandemic employed native workers 7% have lost their job, whereas 13% of migrant workers have been laid off.

Under a scenario without discrimination, the probability of job loss should be equal across workers after controlling for sociodemographic factors and occupation, industry, time, and (data demanding) occupation-by-industry fixed effects (I will discuss the issue of unobserved heterogeneity, such as motivation, below). However, comparing migrants with otherwise similar natives who work in the same industry and carry on the same profession—analogous to a standard difference-in-differences model—shows that migrants face substantially higher firing probabilities. Compared to German native workers, employees with a migration background are, on average, 4 percentage points more likely to lose their job when otherwise healthy firms are unexpectedly forced to reduce their payroll. This systematic disadvantage rises to a 24 percentage points higher risk of being fired in industries that have been hit hardest by the pandemic, that is, a three-times higher firing propensity among migrants. Moreover, I find no systematic difference across the groups regarding the probability to be put on short-time work. I interpret this pattern as an indication that firms make more efforts to retain their native workforce, while being less reluctant to dismiss migrant workers. Hence, this study provides empirical evidence that discrimination continues through the professional stages and confirms the hypothesis that firing discrimination—i.e., dismissal not justified by productivity differentials—puts additional pressure on the immigrant workforce in times of crisis. The results further suggest that this discriminatory behavior on behalf of employers is primarily due to animus against migrant workers.

The findings are robust to a number of alternative operationalizations, sub-sample analyses, and sensitivity checks. Importantly, I find no evidence of systematic sorting of migrants into economically less successful companies—i.e., with a higher overall risk to dismiss workers—after adjusting for individual and occupational characteristics, nor do I observe greater job loss among the self-employed. Both robustness checks corroborate that this analysis is unlikely to suffer from critical selection bias and that migrants are not systematically less productive than native workers.

This paper proceeds as follows: in the subsequent section, I summarize the relevant literature and derive a theoretical model of discrimination in hiring vs. in firing situations. Moreover, I discuss recent studies highlighting inequalities in the wake of the COVID-19 pandemic. In Section 3, I describe the two data sources and provide information on the institutional setting in Germany and the general effect of the pandemic on the economy. Section 4 describes the identification strategy to assess discrimination in a firing case. Section 5 presents the empirical results and robustness checks; Section 6 concludes.

## 2. Discrimination during the economic downturn

### 2.1. Evidence on hiring discrimination

In the past, discrimination research has made considerable progress in highlighting unjustified differential treatment of ethnic and racial minorities in many countries. Apart from research using observational data [25, 26], field experimental evidence in the form of correspondence tests (sending fake applications to real job advertisements [27]) has repeatedly demonstrated that employers systematically prefer native in-group members over minorities in the recruitment process [16, 28–32]. Noteworthy for the context of this study, Baert et al. [33] show that hiring discrimination varies with labor market tightness. That is, in occupations in which vacancies are difficult to fill, applicants with a foreign-sounding name have a similar callback probability as applicants with native-sounding names.

This extensive and growing empirical evidence on the widespread prevalence of hiring discrimination, however, faces three important challenges: first, the hiring of immigrants typically comes with additional considerations, for instance related to work permit acquisition or dealing with regulations on the preferential treatment of natives, established in many countries. Such administrative confounders complicate research on hiring discrimination, whereas firing scenarios do not entail comparable differential treatment of migrants and natives as stated in the law.

Second, while being hired obviously represents a crucial steppingstone, focusing on job entries may conceal discriminatory patterns in later employment-related decisions, such as promotions and eventual firing. In a recent meta-analysis, Quillian et al. [16], for instance, show that there is a difference between being invited for a job interview (the classical outcome of correspondence studies) and actually being offered the job: majority applicants receive not only about 50% more callbacks on average but also almost 150% more job offers than minority applicants. Auer and Ruedin [15] further demonstrate that approximately half of the immigrant workforce in Switzerland report incidents of discrimination at the workplace (see also Larkey [14] for qualitative evidence). Differential treatment is typically not limited to migrants but extends to ethnic and racial minorities in general. Using a dataset of personnel records from a large retailer in the U.S., Giuliano et al. [34] show that the manager–employee racial match (e.g., both White or both Hispanic) is associated with a lower dismissal probability and a lower likelihood of quitting the job. Moreover, this own-race bias also results in greater chances of being promoted when employees and managers share the same race.

Third, (experimental) evidence at the *hiring stages* faces the challenge to identify which processes are responsible for the minority disadvantage [35]: either employers may plainly reject individuals with a migration background or other ethnic and racial minority traits and thus prefer hiring a native worker (*taste-based discrimination*, introduced by Becker [10], or employers may be more uncertain about the true productivity of minority applicants than about that of native ones (*statistical discrimination*, introduced by Phelps [11] and Arrow [12]). The latter type of discrimination is typically regarded as economically rational, whereas taste-based discrimination is driven by animus and xenophobic attitudes and would require substantially different policy responses [35].

Hence, in the standard hiring scenario, discrimination—i.e., differential hiring decisions that cannot be explained by productivity-related individual traits—is typically defined as the result of either statistical or taste-based discrimination, or both. A basic model of the migrant–native *hiring gap* can be described as the following two-term function (similar to Carlsson et al. [36]):

$$P(H_{ij0})=(V_{ij0}^{r}*\eta_{i})+(V_{ij0}^{u}*\mu_{i})$$ (1)

where the hiring propensity *H* of individuals with group-specific traits *i* = (migrant, native) applying for job *j* in period 0 is a function of revealed productivity-relevant characteristics *V^r^* (educational degree, age, etc.), multiplied by a group-specific, unknown discount factor driven by employers’ taste *η* = {0,…,1}, and initially unobserved productivity-relevant characteristics *V^u^* (motivation, adaptability, etc.), amended by a group-specific, unknown discount factor *µ* = {0,…,1}, representing employers’ uncertainty about the candidate’s true productivity. For simplicity, I limit the analysis to two groups, migrants and natives, for which Eq 1 would also allow for positive discrimination, that is, preferential treatment of migrant workers. Based on the empirical evidence, however, we can assume *η*,*μ_native_*≥*η*,*μ_migrant_*.

### 2.2. Revealed productivity and firing discrimination

Once hired, initially unobserved productivity-relevant characteristics are more and more revealed because employers learn about their workers’ productivity, such that the relative importance of the employers’ uncertainty decreases vis-á-vis the group-specific taste-based discount factor. The concept of employer learning constitutes an important aspect of the economic literature on wage setting and labor mobility [37, 38]. In general, there is evidence that employers learn quickly about the true productivity (as a combination of observed and initially unobserved factors) of their workers [39], albeit quantifying this is empirically challenging. Lange [40], for instance, shows that employers’ expectation errors at the hiring stage decline by 50% within 3 years.

There is mixed evidence whether employer learning is heterogenous across occupations and skill sets [41, 42 vs. 43]. However, any potential differences are accounted for after adjusting for education and occupation-by-industry fixed effects (see Section 4). More important for this study is whether employers learn differently about the productivity of migrant versus native workers. Pinkston [44], for instance, finds that employers in the US statistically discriminate against Black labor market entrants at the hiring stage because of hard-to-observe characteristics, but that employers learn faster about the productivity of Black workers, thereby decreasing the importance of this group-specific heterogeneity for earnings. Qualitative evidence corroborates this finding [45], showing that US employers update their stereotypes with regard to single Black workers, but not about Black males as a whole, implying that employer learning exists and reduces discrimination for co-workers, but is of little help for new hires.

I follow the seminal work by Altonji and Pierret [46] and Altonji [47] and hypothesize that migrant and native co-workers converge over time with regard to their revealed productivity because of employer learning. It follows, that employers learn faster about their migrant employees if hiring discrimination exists, as supported by previous studies [44, 45]. As a consequence, in a *firing scenario* triggered by an exogenous shock—such as the COVID-19 pandemic—the dominant and potentially only remaining group-level discount factor of skills is an employer’s taste or stereotype about a certain group *ηi*, thus Eq 1 narrows to

$$\begin{array}{c} P(F_{ij1})=(V_{ij0}^{r}+V_{ij0}^{u})*\eta_{i}\\ =(Z_{ij1}^{r}*\eta_{i}) \end{array}$$ (2)

That is, *firing propensity* in the subsequent period is a function of all revealed productivity-relevant characteristics $Z_{ij1}^{r}$ (education, motivation, etc.) times the group-specific discount factor *η_i_*, i.e., taste. Note that the unobserved productivity component is unlikely to be 0. However, based on the extant literature it is plausible to assume that it converges to 0 over time, so that the remaining heterogeneity in productivity discounting can be mainly–if not fully—attributed to taste-based discrimination. In this study, I will use the exogenous shock of the COVID-19 pandemic that forced firms to lay off workers to estimate the extent of (taste-based) discrimination in the German labor market.

Could it be that migrants are generally less productive than natives? In other words, are migrants just the “first in line” when employers have to dismiss workers? While it cannot be completely ruled out, the empirical literature clearly suggests that migrant workers are, on average, no less productive than their native co-workers; a higher foreign share is often even associated with better firm performance [48, 49]. Moreover, I here provide evidence why the existence of systematically underperforming migrants is unlikely: first, I show that the migrant–native firing gap does not exist for self-employed respondents, indicating that migrants are equally productive than natives at least among the self-employed. Second, migrants do not seem to self-select into “bad” firms (i.e., companies with a higher overall layoff propensity), ruling out that any migrant–native gap is driven by sorting at the company level. Third, economic rationale and employer learning implies that unproductive workers—independent of their migration background—are crowded out over time, so that the importance of the uncertainty discount factor decreases relative to the taste discount factor. This is particularly the case when an economy is not short on labor supply, so that less productive workers are easy to replace. This has been the case in most industries of the German economy prior to the COVID-19 pandemic. The Federal Ministry for Economic Affairs [50] identified STEM and health-related occupations to be affected by labor shortage in some regions of Germany. DeVaro et al. [51] test this economic rationale for promotions. To account for variation in labor supply, I adjust all models with the industry and month fixed effects as well as their interaction term, as explained in Section 3.3. These fixed effects account for differences in the pre-crisis level, such as unemployment. For instance, some industries might naturally have higher levels of turnover, and thus job loss. Moreover, the interaction term generates a very fine-mesh layer of 150 different occupation-by- industry jobs. That is, they also account for within- industry differences across jobs, which is key for identification. Companies of a given industry typically employ different jobs, to which migrants and natives may select themselves differently. Moreover, employment protection might differ across industries and occupations in Germany, for instance, a works council (if existing) has to be involved in firing decisions, which makes adjusting for occupation-by- industry effects even more important. Fourth, in the empirical analysis, I further adjust for a series of key individual characteristics that approximate productivity both directly (e.g., earnings, education, and age) and indirectly through contractual status (part-time or fixed-term contract, which also relates to employment protection) and through the respondent’s perception of being overqualified for the assigned tasks. These variables should account for any potentially remaining productivity differentials.

### 2.3. Inequality during the economic downturn

To the best of my knowledge, there is no study to date that has specifically investigated *firing discrimination*. Yet, there is a small but—due to the current pandemic—fast-growing body of literature on general employment patterns of different groups during recessions. Existing evidence mostly reports increasing inequalities between natives and ethnic and racial minorities during an economic downturn but not necessarily as a result of discrimination [17]. Going back to the Great Recession in the U.S., Couch et al. [52] find that the odds of job loss were significantly higher among Black and Hispanic communities. They also report the same pattern for the 1990s and early 2000s [53]. Cavounidis [54] provides descriptive evidence from Greece that the migrant unemployment rate in Greece prior to the 2008 financial and economic crisis had been lower than that of natives but surpassed native unemployment after the onset of the crisis. A similar pattern is reported by Gandini and Lozano-Ascencio [55] for migrants from Latin America and the Caribbean in the U.S. Using data from the European Labor Force Survey, Cebolla-Boado et al. [56] demonstrate a widening gap between migrants’ and natives’ returns to education during the Spanish recession of 2008 albeit with considerable heterogeneity depending on the origin country. In contrast, Paggiaro [23] finds no difference in job loss between migrants and natives in Italy during the economic downturn of 2009 once observable characteristics predicting unemployment are accounted for. Using administrative data and yearly bankruptcy filings in Norway, Bratsberg et al. [24] observe that low qualifications, precarious tenure, and sorting into firms with a high risk of downsizing are the main reasons why immigrants are more likely to be affected by job loss than natives. This means that the increasing inequalities between migrants and natives during the economic downturn may not be driven by firing discrimination but by individual characteristics and sorting into specific industries and firms that make migrants more susceptible to job loss.

Several studies have investigated the economic crisis in the wake of the COVID-19 pandemic. Lippens et al. [57] use Belgian online survey data collected in March 2020 to show that migrants–and other vulnerable groups–expressed disproportional fear of negative career impacts due to the COVID-19 crisis. Their forecast has been largely confirmed. Several studies using data from the Current Population Survey in the U.S. find that Blacks were only slightly more affected during the onset of the pandemic until April 2020, suggesting that favorable occupational sorting might have mitigated large employment gaps, whereas Latins suffered severely from unemployment [5–7]. Other recent studies look at individual layoffs across occupations with different exposure to the crisis (e.g., whether a job can be done from home; see below) and report more negative effects for immigrant workers [9, 58, 59]. In the UK, Hu [60] shows that Black, Asian, and minority ethnic (BAME) migrants are more likely to experience job loss than White British. This study does not rely on repeated cross-sectional observations during the pandemic, nor does it make use of differences in the industry-specific size of the shock over time. Nonetheless, it reveals several interesting insights at the aggregate level: first, crisis-related layoff probability seems to follow an ethnic hierarchy [61, 62]: the layoff rate for White migrants is similar to that of White British, whereas the layoff rate among BAME migrants is more than twice as high. Second, furlough is more common among White British than among non-White migrants, which suggests that employers prefer to try and keep their White British workers, while fewer efforts are made for ethnically distant migrants.

Importantly, these studies share the limitation that they do not cleanly separate firm-level behavior from immigrant sorting. Hence, none of these studies explicitly investigated the role of discrimination during the economic downturn, as differential effects could be either driven by the self-selection of migrants into specific industries and occupations (sensitive to economic shocks), or by discriminatory behavior of employers, or both. This study contributes to the literature by combining detailed individual data with the exogenous shock of the economic downturn during the COVID-19 pandemic. These two aspects allow for a more accurate measure of heterogeneous firing probabilities across groups in response to an exogenous shock, while accounting for occupational sorting.

## 3. Data and background

The empirical analysis of firing discrimination is based on comprehensive survey data collected in a repeated cross-section in Germany 2020 that is representative for the German resident population. I combine this information on individual job losses across all industries of the economy with official aggregated statistics on monthly transitions into unemployment as an industry-specific measure of the magnitude of the economic shock.

### 3.1. Repeated cross-sectional representative survey data

I use online survey data on N = 11,440 individuals. In a repeated cross-section, this data comprises 17 survey waves fielded (bi-)weekly between April and December 2020. Due to a change in data collection funding, no waves were fielded in September and October 2020. As a sensitivity check, I provide results of the main specification when restricting the observation period to end in August. S7 Table shows that the results are robust to this shorter time frame. The survey was designed to gather information on various topics related to the pandemic including more sensitive questions such as triage, and took approximately 18 minutes to answer. Importantly, I exclusively use questions about the employment situation and general demographics that were asked at the beginning of the questionnaire, thus ruling out any bias, such as satisficing or social desirability that may have been caused by more sensitive subsequent questions. The sample was drawn online by a survey institute, aiming for representativity of the German adult working age population (18 to 65) with respect to age, gender, and education in every wave (N*wave* = [553;1078]; N*mean* = 724; respondents could only participate once). All survey participants gave their consent to participate in the anonymous online survey. After being informed about the responsible research institute, the purpose of the study, that their anonymously answers would be used for scientific purposes only, and their right to quit the survey at any time, respondents agreed to participate. Completion of the entire questionnaire was considered to indicate participant consent. S1 Table provides detailed information on the sample periods and the targeted sample shares. The respondents substantively resemble the German population with regard to key demographics, including gender, age, and education, and even in terms of occupation. Moreover, the reported within-occupation layoff shares–the main outcome–are very close to the official statistics, which further raises the confidence that the results of this study are generalizable to the German population. Note, however, that such online-recruited samples rarely achieve full representativity of the resident population, so that with few exceptions (gender and in parts education and profession) standard one-sample tests of proportion suggest that the sample means significantly differ from the population means. To assess whether misrepresentation biases the results I re-estimate the main model using post-stratification weights in Section 5.2. below.

#### 3.1.1. Occupation, industry, and job loss

To capture the effect of the economic shock in the wake of the pandemic, I restrict the sample to individuals who have been employed as of March 1, 2020, i.e., before companies reacted with mass layoffs to the pandemic. (Question wording translated from German: “If you think back to the time before the Corona crisis, right before March 1, 2020, were you gainfully employed at that time?”.) These respondents were subsequently asked to report any changes in their employment situation since March. Specifically, the main outcome of this study—*layoff*—is defined as 1 if a respondent reported to have lost his or her job since March (independent of whether the person has found new employment in the meantime) and 0 otherwise. This definition does not differentiate between different types of changes in employment status (job loss and transition into unemployment, job loss and new employment, job loss and change of occupation, and termination for other reasons), as it is inherently difficult to identify the determining factors in a complex situation involving economic but also individual crises. Consider, for instance, a situation in which the employer indicated that the pandemic might force her to lay off the worker and that worker, in anticipation of her imminent firing, searches for (and finds) a new job. This person would answer the question in the survey with “changed employer, same occupation” or “changed occupation”. However, in Section 4.2 I will also show that the effects are robust when restricting job loss to the incidents that respondents reported as being a direct result of the pandemic (about 66% of all reported layoffs), and when restricting job loss to respondents who were unable to find a new job (approximately 70%). As an additional outcome, I capture the reported short-time work situation of respondents, that is, whether they have been working at a reduced level of time and pay temporarily since March. In total, 12% of all respondents report having lost their job, and almost 20% report having been on short-time work between March and December. Note that the survey does not capture the exact date of being fired or put on short-time work, which I will discuss in more detail below.

S2 Table shows that the industry composition of the employed respondents, similarly to the sociodemographic information, mirrors the distribution in the German economy. I obtain the occupational position of every individual from two free-text questions on the employment industry and the specific job, respectively, and from information about the respondent’s professional education. This information is then classified according to the official definition of industries [63] and the International Standard Classification of Occupations at two digit level (ISCO-08 with 10 major categories [64]) so that every employee can be assigned to an industry and specific occupation within this industry. The free-text approach to capture occupation and industry follows established procedures [65]. (Question wording: “What professional activity were you engaged in immediately prior to March 1, 2020?” and “In which economic sector/industry/service sector is the company or institution in which you were predominantly active before March 1, 2020?”.) From 11,440 responses, 289 entries for occupations and 1,192 entries for industries were invalid, with the vast majority missing or containing nonsensical information. Additional robustness checks confirm that the main findings are robust to including respondents who indicated that they had lost their job loss but for whom no valid ISCO or industry information was available (Section 5.2). In addition to the industry and occupation classification among those who were employed until 1 March 2020, the survey collects several variables that may predict their firing probability: first, respondents’ subjective assessment of whether they feel under- or overqualified for their job (henceforth labelled as “feeling overqualified”; question wording: “To what extent did the requirements of your job match your skills?”), which functions as a proxy for job satisfaction [66], individual productivity (Ma et al. [67] report a positive relationship between perceived overqualification and productivity, while Lee et al. [68] find mixed results), and a number of additional aspects related to the employee-workplace relationship, such as turnover intentions (for an overview, see Erdogan and Bauer [69]). Second, in all models I adjust for the logged household income of respondents. While this is a rather crude proxy, it should capture part of residual heterogeneity related to job quality. Third, respondents were asked to indicate their specific employment status before March, i.e., whether they were employed on a full- or part-time basis and whether their contract was temporary or permanent (Question wording: “Were you employed full-time or part-time prior to March 1, 2020?” and “Was your employment contract before March 1, 2020 temporary or permanent?”), assuming that both fixed-term and part-time employment is more susceptible to job loss due to lower firing costs and differences in employment protection [70–73].

#### 3.1.2. Migration background

The definition of the main independent variable—migration background—follows the official definition by the Federal Office of Statistics [74]. For simplicity, I use the terms migrant and people with a migration background interchangeably. The migrant indicator equals 1 if the respondent or at least one parent was not born in Germany. The exact definition by the Federal Office of Statistics [74] bases migration background on citizenship at birth. For simplicity, the survey asks for the country of birth. Note that the survey was administered in the German language only, which likely explains the underrepresentation of migrants (c.f. S2 Table; S3 Table shows the origin countries of the survey respondents with a migration background) and may also have led to an underrepresentation of workers not proficient in German. Given that language proficiency is an important predictor not only of labor market participation [75, 76] but also of discrimination [77, 78], any observed discriminatory pattern among the survey respondents is plausibly a lower-bound estimate of the total extent of discrimination in the German labor market. In addition, this definition of migration background is agnostic about citizenship and ethnic or racial traits. In other words, respondents need not possess any visible traits or a non-German citizenship that would make them easily identifiable as individuals with a migration background, so that we would expect any differences between migrants and natives to be a conservative estimate of potential discrimination against more visible minority groups [79]. In Section 5.2, I test firing discrimination for two alternative migrant definitions.

Taken together, this data allows me to estimate whether migrants are more likely to enter unemployment since March, which would—conditional on the industry, timing, and occupational role—provide a strong indication of *firing discrimination* by German firms. Translated into the established dichotomy of statistical vs. taste-based discrimination in the extant literature, firing discrimination cannot be explained by employer uncertainty about the workers’ true productivity (as in the case of *hiring* situations). This is because these workers have gradually proven their true productivity so that systematic differences are predominantly attributable to taste-based discrimination by employers with anti-immigrant attitudes.

### 3.2. Employee protection in Germany

To better assess whether firing patterns are discriminatory or a reflection of objective differences between workers I briefly summarize the institutional background of employee protection in Germany. In general, workers enjoy relatively high levels of protection and discrimination is theoretically prohibited. Still, statutory differences across groups exist. The main aspect among regulations that would allow for differential firing is collective bargaining of certain industries and professions. In 2019, 44% of workers in Germany have been employed under a collective bargaining agreement [80]. Yet, workers of the same employer are typically required to be treated equally, even if not everyone is employed under a collective bargaining agreement (so-called “equality agreement” [81]). That is, even if the migrant workers in a firm were not represented by a labor union, they would enjoy collective agreement rights, as long as their native co-workers were unionized. To account for remaining differences (e.g., possible sub-contractors), I adjust for occupation-by-industry fixed effects. Further, within occupations within industries, the type of employment contract can play a role, in particular whether a contract is permanent. In addition to controlling for easier firing of workers with a fixed-term contract, I adjust for part-time contracts, which are–in some cases–also easier to dissolve.

Eventually, within contract types within occupations within industries, individual characteristics determine who of two otherwise similar workers can be fired first. The Dismissal Protection Act [82] regulates that layoffs should be “socially acceptable”. If a company employs more than ten workers and if two or more workers are perfect substitutes (same tasks), “social selection criteria” apply. That is, who is to be fired should depend on the age, employment duration in the firm, child support obligations, and disability. The data does not capture sensitive information on individual disability. Yet, this social selection criterion arguably does not occur often enough to affect general firing patterns. Of the remaining three, age and the number of children are directly observed and adjusted for in the estimations. The questionnaire does not, however, measure employment duration (with the same employer). That is, if employment durations of migrants were systematically shorter than for same-age native co-workers (with the same contract type, performing the same occupation, in the same industry), the estimates could be biased. I address this omitted variable concern using complementary data on within-company employment durations from the European Labor Force Survey [83]. Second, I investigate the effect within age groups, and, third, perform two tests on the importance of unobserved selection [84, 85]. Fourth, I show patterns of labor court decisions in Germany over time as descriptive evidence on the perception of unjust firings during the pandemic. Each of these additional analyses, which are discussed in detail in Sections 5.2. and 5.4., respectively, suggest that unobserved heterogeneity in employment durations is, if anything, marginal, and unlikely to affect the main results.

Overall, considering the objective criteria that may justify differential firing (occupation-by-industry fixed effects, contract types, and social selection criteria) implies that such a model also accounts for differences between “core” and “marginal” workers. This categorization of workers within a firm into more privileged essential workers and more “expendable” ones (who are also less protected) constitutes a key aspect of labor markets in industrialized countries [86, 87].

### 3.3. Industry-specific effects of the pandemic

In times of normal economic activity, changes in employment status may not reflect firing discrimination but rather result from natural labor turnover, while labor shortages may even conceal discriminatory firing practices. In times of crisis, however, otherwise healthy firms are suddenly forced to dismiss part of their workforce on short notice and in large numbers.

Recent studies have provided categorizations to assess the heterogeneous impact of the COVID-19 pandemic and related containment efforts across different industries and occupations. They focus primarily on the ability to work from home, such as Dingel and Neiman [88], who calculate the share of “teleworkable” jobs for each industry in the U.S. Similarly, Koren and Peto [89] measure the percentage of workers in an industry that is exposed to face-to-face interactions (with co-workers and customers) and hence most affected by social distancing policies. Basso et al. [90] expand this approach to a general classification of jobs according to their contagion risk. Alon et al. [91] add to this a categorization of whether an industry is considered “critical” (e.g., pharmacies). Based on such classifications, several studies find that inequality in terms of wages and working hours along gender [58, 92], education [5, 92], and migration background [5, 58, 59] increases during the pandemic. While such measures are suited to evaluate the industry-specific impact of the pandemic in the future, they need to approximate various dynamics related to the overall economic shock, changes in consumer demand, containment measures, etc. Moreover, these indices are less suited to assess discrimination, given migrant sorting is not confined to industries but also takes place at the occupation level within industries. Observing both sources for migrant sorting is essential to estimate heterogeneous effects of the pandemic on different groups. For instance, an office clerk in manufacturing may have had the possibility to work from home, while an assembly line worker in the same company had not. Hence, it is important to know the occupation of the respondent. Vice versa, the same office clerk in manufacturing may have had a different layoff probability than, for instance, an office clerk in service, because industries were hit by the economic shock to different extents. Hence, it is also important to know the industry in which the respondent has been employed. Accordingly, the approach chosen here to adjust for occupation-by-industry is arguably better suited to address sorting.

I draw ex-post on official registry data of 2020 to model the heterogeneous consequences of the COVID-19 pandemic across different industries of the German economy. To capture the magnitude of the economic pressure on firms, I use official statistics provided by the Federal Employment Agency [3], reflecting the cumulative monthly numbers of newly registered unemployed in 2020 relative to the same industry -months in 2019 (again based on the official definition of industries [63] by the Federal Office of Statistics 2021a).

**Fig 1:**
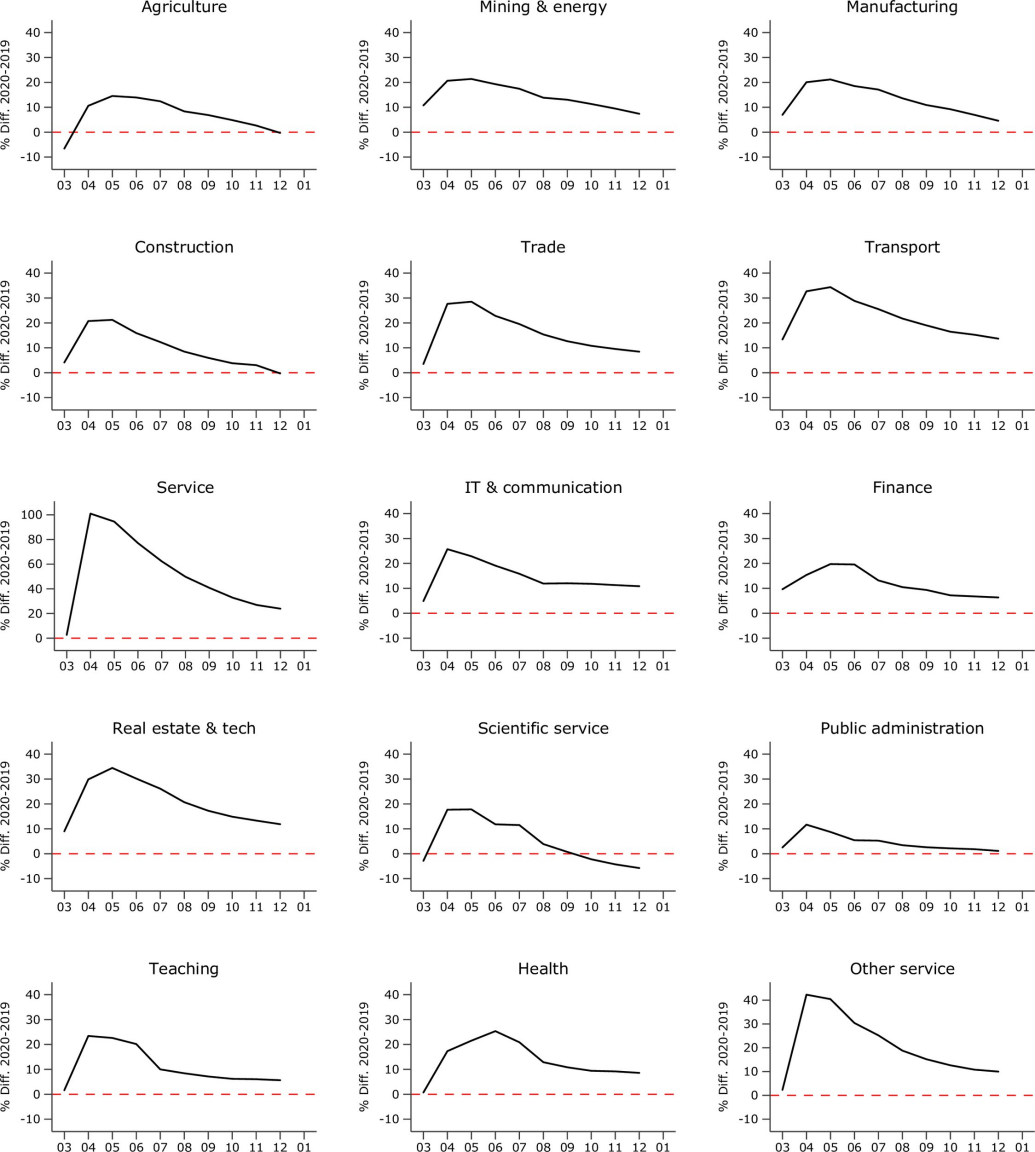
Economic pressure across industries and time. Note: Fig reports the cumulative difference in layoffs between 2020 and 2019 in percent as reported by the official statistics. Industry definition following Federal Office of Statistics (2021a). Source: Federal Employment Agency [3], own calculations.

For instance, in March 2020 the Employment Agency reported that 12,840 workers in the service industry had lost their job. Comparing this number to 12,505 entries into unemployment in March 2019 results in a mild increase of 2.7% at the very onset of the global pandemic. In April 2020, the number peaked at 35,348 newly unemployed, a 208.2% increase compared to April 2019 (11,468) and a cumulative change in unemployment—the main *shock* indicator—of (12,840+35,348)/(12,505+11,468)~101%. Hence, the magnitude of the economic shock for a service industry worker who answered the survey in July 2020 is derived from the cumulative changes in unemployment from March to July 2020 relative to the same period in 2019. This cumulative measure is meaningful given that firing decisions by firms are path-dependent in the sense that they depend not only on the current economic situation but also on developments in previous periods. Fig 1 shows the cumulative magnitude of the economic shock for each industry. The full matrix of monthly changes across all industries is provided in S4 Table and visualized in S2 Fig. As a robustness check, I also estimate the probabilities of being fired/put on short-time work in a lagged model, i.e., in which cumulative unemployment changes are measured until the month prior to the time of survey (see Section 5.2).

**Fig 2:**
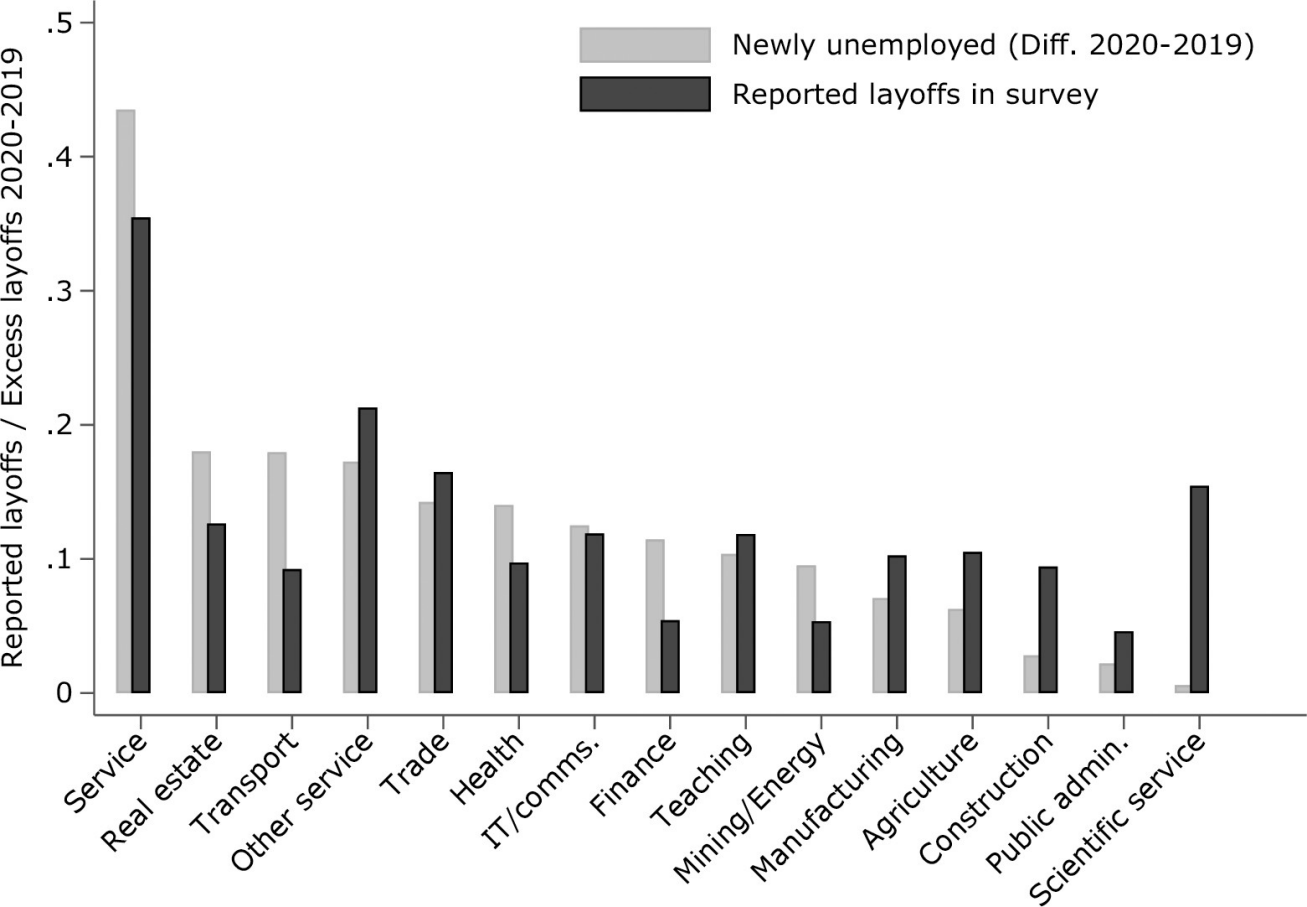
Reported layoffs and official statistics across industries. Note: Fig compares layoffs as reported by the survey respondents over time and across industries and compares them to official statistics. Source: Federal Employment Agency [3], own calculations.

In the wake of the pandemic, every economic industry reported increased numbers of layoffs in April and May 2020 compared to the reference month in 2019, and most industries of the economy had persistently higher numbers of newly unemployed until July, before a slight recovery started. Importantly, the *magnitude* of the economic shock varied considerably across industries [9]. The service and tourism industry, but also transport, trade, and real estate services were hit particularly hard, whereas public administration and the finance industry reported relatively mild increases in monthly transitions into unemployment (c.f. S4 Table). The average deviation in newly unemployed from the 2019 reference period per industry is shown in Fig 2. Accordingly, for the entire period between March and December 2020 the average number of newly unemployed individuals has been about 8% higher compared to the 2019 reference period. The shares derived from the individual layoffs reported in the survey (black circles) are remarkably close to the official statistics (gray diamonds), especially considering the small sample sizes in some economic industries (N*min* = 38 in agriculture, N*max* = 1,023 [945] in manufacturing [health], N*mean* = 452), which further supports the representativity assumption.

## 4. Identification

The shock nature of the COVID-19 pandemic, accompanied by measures taken to slow down its spread, provides a natural experimental setting in which otherwise healthy firms are suddenly forced to dismiss their workers or, if possible, to put them on short-time work. Normally, differences in job loss propensities would be attributed to migrants and natives selecting themselves into different types of occupations [18–21], so that we need to adjust for this sorting.

The exogenous shock resulting from containment measures in the spring of 2020 indiscriminately affected all firms in Germany. If we assume that employers are rational actors who hire and retain workers if their demonstrated productivity is in line with the expected productivity, any productivity-related unobserved factors would be non-systematically associated with individuals within a given industry and occupation. In contrast, if we presume that immigrants in the German labor market are subject to hiring discrimination [28, 93, 94], migrants who are employed would have to possess some trait—such as motivation or a particular skill—that makes them relatively *more productive* to outperform the native labor supply during the hiring stage. Hence, we can estimate the (lower-bound) effect of migrant status on job loss—i.e., firing discrimination—with the following model:

$$\begin{array}{c} Y_{isjmr}=\alpha +\lambda \;{migrant}_{i}+\gamma \;{shock}_{sm}+\\ +{x\prime }_{i}\beta +\delta_{s}+\delta_{j}+\delta (s*j)+\delta_{m}+\delta_{r}+\epsilon_{isjmr} \end{array}$$ (3)

where *Y* denotes the layoff probability for individual *i* as a function of *migrant* status, the extent of the industry- and month-specific economic *shock*, individual- and contract-related controls *x’*, and a number of fixed effects capturing variation in respondent’s industry *s* and occupation *j* as well as an interaction of industry and occupation fixed effects to adjust for variation in job-level responses across industries [58, 92]. Moreover, I adjust for the survey month *m* to account for temporal differences (e.g., increased hiring of construction workers in spring) and the respondent’s location at the state level *r*.

As mentioned above, the impact of the economic shock varied across industry and time, requiring the inclusion of *shocksm* above. I add to the baseline Eq 3 an interaction term of migrant status and the industry- and time-variant extent of the economic crisis. The latter yields the estimator comparing migrant workers’ layoffs to the ones of natives within industries that were hit to different extents by the pandemic:

$$\begin{array}{c} Y_{isjmr}=\alpha +\lambda \;{migrant}_{i}+\psi \;{shock}_{sm}+\tau ({migrant}_{i}*{shock}_{sm})+\\ +{x\prime }_{i}\beta +\delta_{s}+\delta_{j}+\delta (s*j)+\delta_{m}+\delta_{r}+\epsilon_{isjmr} \end{array}$$ (4)

where *τ* captures the heterogeneity of different crisis intensities between migrants and natives, analogous to a standard difference-in-differences model. Importantly, the extensive set of occupation, industry, and industry-by-occupation fixed effects means that we compare migrants vs. natives within the same industry that is hit differently at different points in time in 2020. Hence, we can also rule out that certain unobserved differences between migrants and natives in a given industry exert estimation bias. For instance, industries differ in their level of unionization and formalized employee protection, as discussed in Section 3.2. above.

**Fig 3:**
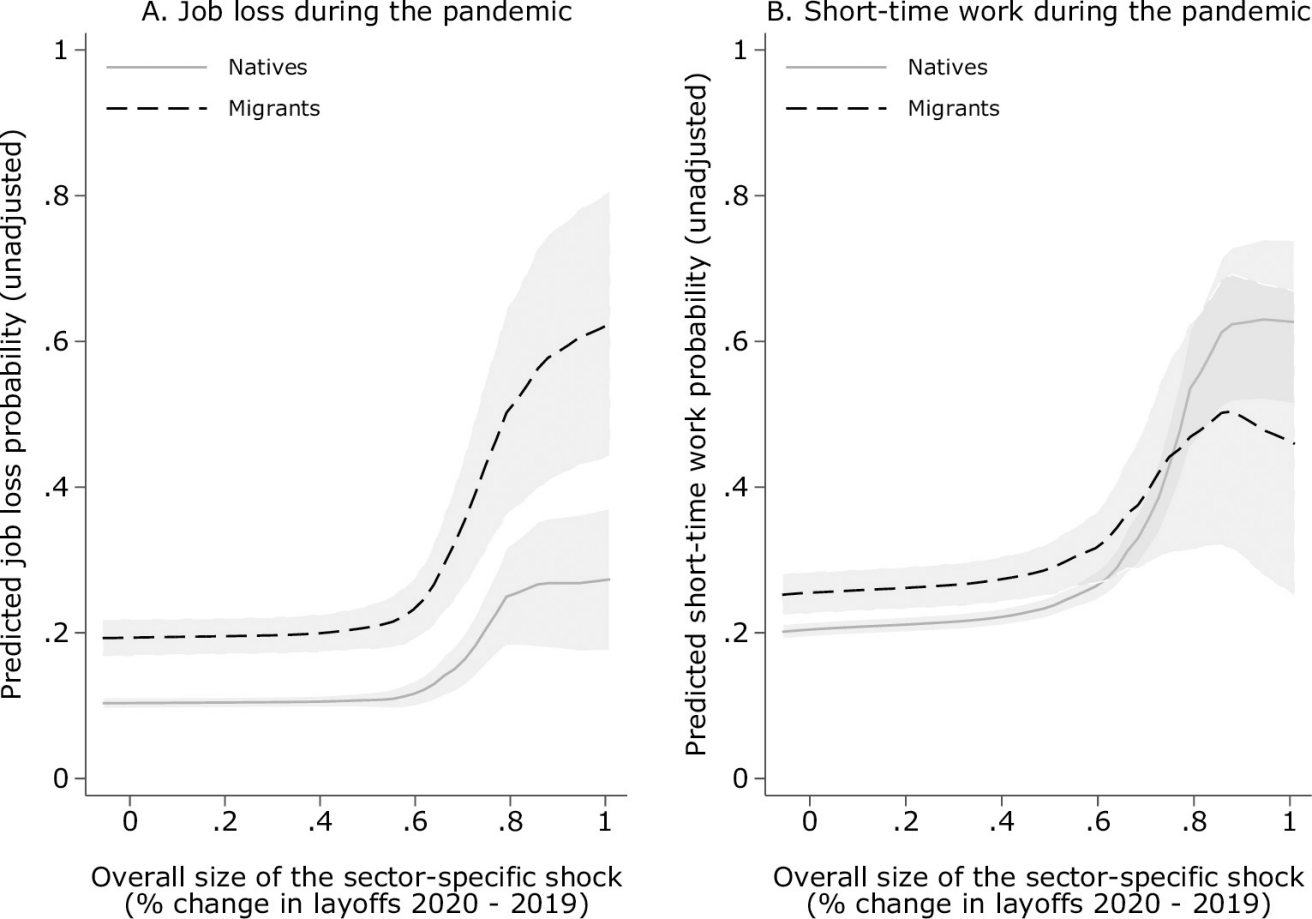
Raw difference between migrant and native layoffs. Note: Fig compares unadjusted layoff and short-time work propensity as reported by survey respondents with a migration background and without by the magnitude of the industry-specific economic shock (measured by the difference in newly unemployed between 2020 and the reference month in 2019). Local polynomial regression (bw = 0.2). Strongest cumulative change observed in service industry (+101% unemployed compared to 2019). S1 Fig shows the corresponding trends for the restricted sample of respondents surveyed in April and May (onset and peak of the shock). Source: Federal Employment Agency [3], own calculations.

Panel A in Fig 3 provides the raw differences in dismissal rates for migrants and natives across the magnitude of the economic shock in the industry in which the respondents have been employed. As an alternative outcome, I estimate the effect of migrant status on the probability to be put on short-time work (Panel B). Short-time work being a crisis measure, it is not possible to establish a comparative measure with 2019 as the reference year. The Federal Employment Agency also does not provide consolidated monthly data on the number of short-time work take-up across industries. Hence, I will maintain the change in layoffs as the preferred industry-specific measure of the economic shock. Overall, migrants consistently report higher layoff rates than natives, independent of the magnitude of the economic shock (dashed black line in Fig 3, Panel A). Moreover, in the unadjusted setting, reported layoffs among natives (gray solid line) in the hardest-hit industry-months (up to 100% excess unemployment compared to 2019) double, whereas migrants report about three times as many layoffs in these industry-months compared to the baseline propensity of about 20% in the least-hit industries. With regard to transitions to short-time work, the picture looks different: while the overall probability to be put on short-time work increases with economic pressure, it is the native group that seems to be more likely to be put on short-time work than to be fired (for the beneficial effects of short-time work on firm survival, see [95–97]). This could be a first indication that firms are making relatively more efforts to keep their native workforce.

Note that one further reason for defining the shock magnitude as the cumulative change in layoffs is that the survey did not capture the exact date of job loss, so that respondents from later waves may have lost their job already in spring. In fact, the impact of the crisis in terms of mass layoffs peaked in every industry between April and May 2020 (c.f. Fig 1). This inaccuracy can, by construction, not affect the measure of the economic shock (as it is based on the official cumulative monthly job loss numbers), and it is reasonable to consider survey-reported layoffs as well as the cumulative response to the crisis up until the respective survey month. However, it may lead to an overestimation of crisis-induced layoffs in industry-months later in 2020, when recovery set in. That is, for instance, we may wrongly ascribe a job loss reported in September to September (during recovery) although the respondent lost his or her job already in April. This over-reporting might explain the relatively high unadjusted layoff probability in Fig 3 of 10–20% in mildly hit industry-months. Such over-reporting, in turn, may result in an underestimation of the slope of migrant and native layoffs, which are expected to increase with the severity of the crisis.

I approach this potential bias at the lower tail with two robustness checks shown in Section 5.2: first, I re-run the main model but restrict the observation period to April and May 2020, when excess layoffs could only be caused by the onset of the pandemic. This approach trades variation within industries over time for a more accurate picture of the relationship between the shock magnitude and immediate employment outcomes. S1 Fig indicates that the overall layoff patterns for the April/May sample closely resemble the full sample period for both groups. The same holds for the native sample with regard to short-time work but not for the migrant sample, which seems to benefit much less from reduced working hours to avoid job loss during the onset of the pandemic. Moreover, as expected, the slopes for the April/May sample are steeper because of arguably less over-reporting in the less hit industries. Second, I estimate the main specification without month and industry fixed effects and by replacing the month-specific shock measure with the March-to-December cumulative shock for all respondents within a given industry so that the repeated cross-sectional data is treated like one single cross-section. This shows that the overall extent of firing discrimination is not affected by an inaccurate timing of the reported layoffs.

## 5. Results

Table 1 shows the OLS estimates of the effect of migrant status on the probability to be laid off (Columns 1–4) and to be put on short-time work (Columns 5–8). Columns 1,3,5, and 7 provide a parsimonious effect without controls. Models 2 and 6 estimate Eq 3, that is, without interacting the industry-month–specific cumulative magnitude of the economic shock with the migrant indicator. As can be seen, the average probability to report a job loss is 3.8 percentage points higher for migrant workers, whereas the short-time work propensity is statistically insignificantly different from natives. Notably, the results also show only a marginal and statistically insignificant gender gap, whereas recent studies have suggested that women are disproportionately affected given that social distancing impacts industries with high female employment shares [91]. This demonstrates the importance to adjust for industry and occupation fixed effects to account for occupational sorting—in this case of women.

**Table 1:** Heterogeneous firm responses to the economic downturn.

	Layoff				Short-time work			
	(1)	(2)	(3)	(4)	(5)	(6)	(7)	(8)
Migrant	0.053***	0.038**	0.007	-0.008	0.036	0.028	0.074**	0.064*
	(0.016)	(0.014)	(0.026)	(0.026)	(0.023)	(0.024)	(0.030)	(0.032)
Shock		0.014	-0.027	-0.022		-0.050	-0.031	-0.022
		(0.038)	(0.027)	(0.033)		(0.146)	(0.135)	(0.145)
Migrant *×* shock			0.243***	0.243***			-0.205**	-0.191**
			(0.079)	(0.081)			(0.080)	(0.083)
Female		0.011		0.011		0.007		0.007
		(0.008)		(0.008)		(0.011)		(0.011)
Age		-0.011**		-0.011**		0.002		0.002
		(0.004)		(0.004)		(0.003)		(0.003)
Age2		0.000**		0.000**		-0.000		-0.000
		(0.000)		(0.000)		(0.000)		(0.000)
No. of children		0.009**		0.009**		0.004		0.004
		(0.004)		(0.004)		(0.008)		(0.008)
Household size		-0.010		-0.009		-0.010		-0.011
		(0.008)		(0.008)		(0.016)		(0.016)
No formal education		0.000		0.000		0.006		0.006
*Ref*. *= Professional educ*.		(0.013)		(0.013)		(0.025)		(0.025)
Technical educ.		-0.003		-0.004		-0.001		-0.001
		(0.015)		(0.015)		(0.014)		(0.013)
Bachelor		0.029		0.029		-0.035		-0.035
		(0.018)		(0.018)		(0.029)		(0.030)
Master		-0.007		-0.007		-0.034		-0.033
		(0.012)		(0.012)		(0.025)		(0.025)
PhD		0.053		0.054		-0.027		-0.027
		(0.046)		(0.046)		(0.073)		(0.073)
Part-time contract		0.022		0.022		-0.012		-0.012
		(0.013)		(0.013)		(0.020)		(0.020)
Fixed-term contract		0.112***		0.113***		0.015		0.015
		(0.016)		(0.016)		(0.028)		(0.027)
Feeling overqualified		-0.001		-0.001		0.002		0.002
		(0.002)		(0.002)		(0.004)		(0.004)
HH income (log)		-0.056		-0.054		0.080		0.078
		(0.060)		(0.060)		(0.082)		(0.083)
Constant	0.004	0.775	0.008	0.760	0.041	-0.760	0.046	-0.748
	(0.021)	(0.622)	(0.020)	(0.625)	(0.025)	(0.843)	(0.032)	(0.852)
R2	0.067	0.099	0.069	0.101	0.131	0.135	0.132	0.135
Observations	5500	5473	5500	5473	5500	5473	5500	5473
Federal state FE (N)	16	16	16	16	16	16	16	16
Month FE (N)	11	11	11	11	11	11	11	11
Industry FE (N)	15	15	15	15	15	15	15	15
ISCO FE (N)	10	10	10	10	10	10	10	10
Industry*×* ISCO FE (N)	150	150	150	150	150	150	150	150

Notes: Table presents the effect of migrant status on the probability to be laid off (Models 1–4) and to be sent on short-time work (Models 5–8). Models 1,3,5,7 estimate a parsimonious model without controls. Models 4,8 provide the difference-in-differences estimator based on Eq 4. Heteroskedasticity and serial correlation robust standard errors clustered at industry level in parentheses.

* p< 0.10

** p< 0.05

*** p< 0.01. *Source*: Federal Employment Agency (2021), own calculations.

Columns 4 and 8 show the difference-in-differences model of Eq 4. We are particularly interested in the interaction term of migrant status with the magnitude of the economic shock. The coefficient indicates that the layoff probability of migrants is 24.3 percentage points higher for migrants if the company’s industry-specific unemployment doubled in 2020 compared to 2019 (i.e., an unemployment increase of 100%). At the layoff sample mean of 8% this translates into a three times higher *average* layoff probability for migrant workers. The mean layoff is lower compared to the unrestricted sample layoffs as shown in Fig 1, due to sample restrictions discussed in Section 3. In contrast, the same increase in the economic shock is associated with a 19 percentage points *lower* likelihood of migrants taking up short-time work (Column 8, line 3). All models control for an extensive battery of fixed effects and individual controls—most importantly on occupation and industry characteristics. Hence, the results do not necessarily suggest that firing discrimination is larger in harder-hit industries than in less hard-hit ones, but that firing discrimination increases within an industry when excess layoffs increase. As expected, part-time and fixed-term contracts are more likely to be dissolved, whereas income and the perception of being overqualified (as proxies for job-level productivity) have no statistically significant effect. In sum, this corroborates the hypothesis that firms are more willing to retain their native workforce, thus providing substantial empirical evidence for *firing discrimination* in the German labor market.

### 5.1. Non-linear effects

A perhaps more accessible approach is to present a residual measure of the economic shock that does not constrain the form. That is, accounting for possible non-linear effects, I categorize the cumulative change in layoffs into 6 groups ranging from *<*0–9% at 10% intervals until a 50% or more average increase compared to 2019 [98]. Adjusting Eq 4 gives the effect for each magnitude category:

$$\begin{array}{c} Y_{isjmr}=\alpha +\lambda \;{migrant}_{i}+\psi \;{cat}_{sm}^{1\ldots 6}+\tau ({migrant}_{i}*{cat}_{sm}^{1\ldots 6})+\\ +{x\prime }_{i}\beta +\delta_{s}+\delta_{j}+\delta (s*j)+\delta_{m}+\delta_{r}+\epsilon_{isjmr} \end{array}$$ (5)

where every industry-month is assigned to a distinct magnitude category, ranging from no change to 50% or more excess unemployment relative to 2019, *cat* = *{<*0–9; 10–19; 20–29; 30–39; 40–49; *>*50*}*. Note that the categorization is at the industry-month level, so that a specific industry can be in several categories. This ensures that the form-agnostic approach does not just capture the effect across different industries alone.

**Fig 4:**
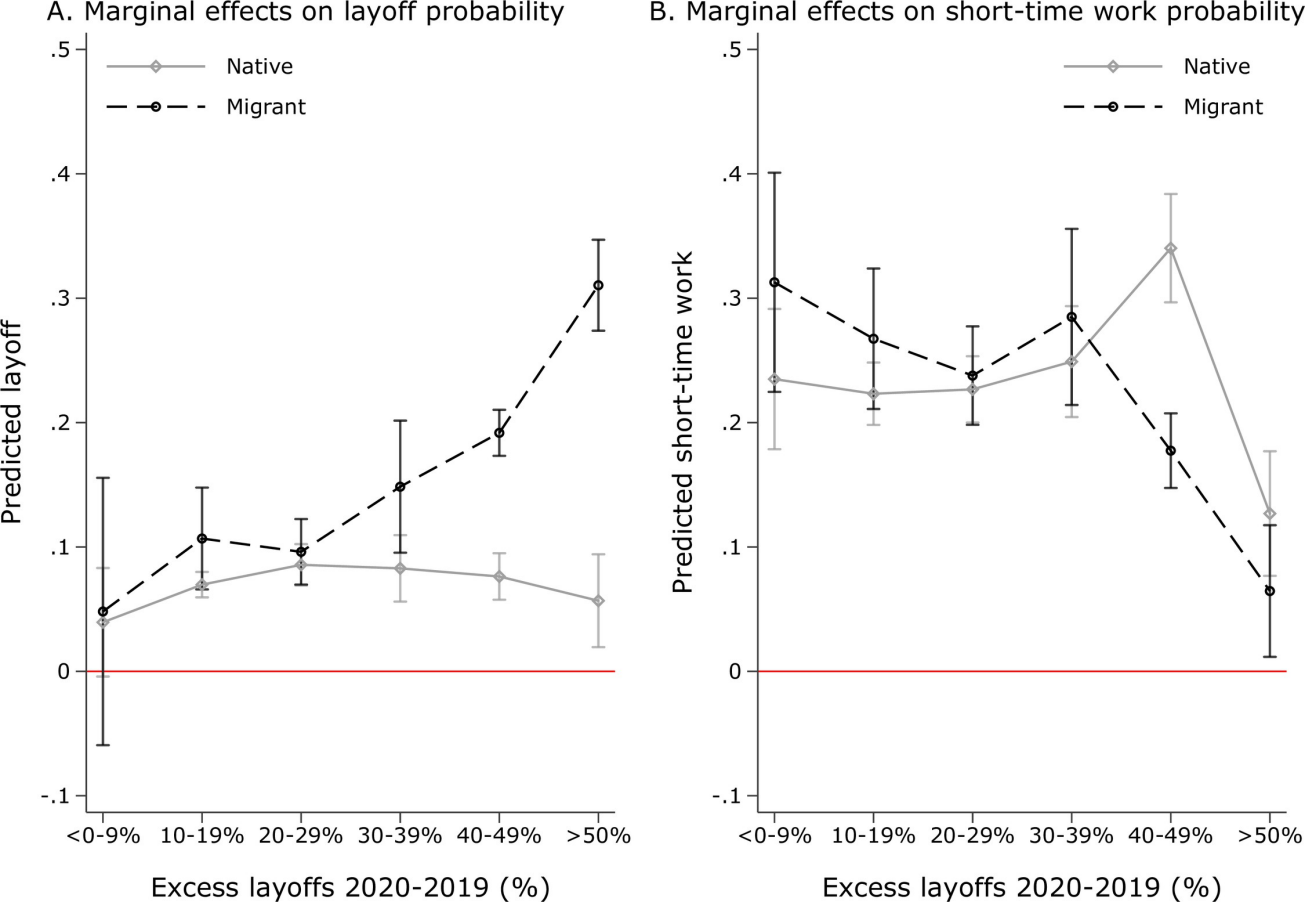
Heterogeneous layoff patterns across crisis magnitudes. Note: Fig provides marginal effects of industry-specific crisis exposure on migrants’ and natives’ layoff/short-time work probability in Panels A and B, respectively. The corresponding regression is presented in S5 Table. Source: Federal Employment Agency (2021), own calculations.

Fig 4 presents the marginal effects on layoff (Panel A) and short-time work probability (Panel B) of the different industry-specific crisis magnitudes applying the industry/shock definitions in Section 3.3. The underlying regression results are shown in S5 Table. Second, migrants are consistently more likely to be fired (Panel A), albeit the difference is small and statistically insignificant in industry-months that were less severely affected by the economic crisis (i.e., an average increase in newly unemployed of 30% or lower compared to 2019). In more affected industries of the German economy, migrants are substantially disadvantaged. Among the most severely hit industries (*>*50%), migrants have a predicted layoff propensity of about 30%, whereas that of natives consistently lies below 10%. Third, the predicted probability of short-time work is less clear-cut at around 20 to 30%. As expected, it is thus higher than the overall predicted layoffs, with a drop in uptake in the hardest-hit industries (in which layoffs dominate). Migrants mark similar patterns as natives; however, the latter group is more likely to be put on short-time work in the most severely affected industries. This finding lends additional support that layoffs and short-time work function as substitutes, with firms—if they can—making more efforts to retain their native workers than to retain their migrant workers.

### 5.2. Robustness

#### 5.2.1. Alternative specifications

Before turning to the sub-group analyses and assessing migrant sorting, I conduct several robustness checks. In S6 Table I first re-estimate Eq 4 by reweighting the sample using post-stratification weights to closer reflect the German resident population in terms of migrant share, gender, age, and occupations (ISCO). The coefficient for layoffs remains substantively unchanged, while the coefficient for short-time work increases in magnitude but turns statistically insignificant. Together, these results indicate that the estimation is not biased by misrepresentation in the sample. Second, I replace the main IV (shock) with a lagged and a monthly measure to test whether the main coefficients in Table 1 are driven by a biased shock definition. Both the lagged measure (cumulative excess unemployment until 1 month prior to the respective interview date) and the monthly model (i.e., a more volatile non-cumulative measure showing the raw change in layoffs independently of previous months) yield coefficient patterns that are very similar to the main specification. S6 Table further replicates the main estimates when amending the main DV—layoffs. The effects remain stable when restricting layoffs to only those incidents in which respondents reported their dismissal to be a direct consequence of the COVID-19 pandemic. As mentioned above, the majority confirmed that their job loss was connected to the pandemic; dropping cases in which respondents indicated otherwise does not affect the findings. Similarly, the patterns remain unchanged when restricting layoffs to those cases in which respondents did not return to (new) employment by the time of the interview. It could have been, for instance, that migrants are just more flexible workers, who can switch jobs faster to respond to changing economic conditions. This seems not the case.

#### 5.2.2. Sub-samples and sensitivity

S7 Table additionally tests the role of sample selection and the validity of defining the shock as a cumulative measure of excess layoffs. As mentioned, we do not know the exact date of respondents’ layoff, which can lead to a mismatch between the timing of job loss and that of the industry-specific economic shock. To confirm that this approach is still valid, I first restrict the respondent sample to the waves conducted between April and May and between April and August, respectively. April and May mark the onset of the pandemic; every industry’s excess unemployment peaked during this period so that pandemic-induced layoffs reported until the end of May can only be driven by these immediate shocks. In other words, this approach trades variation in the shock across industries and months for a more precise match between layoff and shock timing. The results confirm the main findings. The coefficient *τ* is even larger, indicating that the results across the full observation period might reflect a lower-bound estimate of very short-run discriminatory patterns (which is also indicated in S1 Fig). S7 Table corroborates the overall trend when restricting the sample to the time until August, that is, before the temporary suspension of the survey. In addition, replacing the cumulative monthly shock measure with a time-invariant indicator capturing the cumulative excess layoffs at the industry level between March and December 2020 (relative to 2019) for all respondents (independent of the interview date) produces substantively identical results.

Eventually, Columns 4 and 5 provide estimates for the main sample and when including respondents who indicated that they had lost their job but for whom the occupation or industry could not have been identified from the free-text statements. Therefore, I create an additional ISCO and an industry group, which pools these cases. Obviously, for the two catch-all groups, no information on the number of layoffs exists, hence I exclude the shock variable. Both samples produce similar results, with the overall migrant layoff probability being even larger in Column 6—including non-identifiable industries/occupations. This could further point toward a conservative estimate of firing discrimination if migrants with reported layoffs are more likely than respective natives to be excluded from the final sample.

#### 5.2.3. Omitted variable bias

Next, I assess the likelihood that the estimates are biased by omitted variables. In particular, the survey data does not contain information on the employment duration of respondents in the same company. However, if differences in the employment duration between migrants and natives were systematic, estimates could be biased. To address this concern, I use complementary data of the European Labor Force survey [83] on 250,000 respondents in Germany and show that migrants do not differ significantly from natives in the number of years affiliated with their current employer. More specifically, Column 1 shows that employees do not work for a shorter time in a company if they were born outside Germany, while citizenship seems to play a role (Column 2). However, when combining the two definitions in Column 3, only respondents who were born in Germany but who are not Germany citizens appear to have a significantly shorter employment duration (0.8 years, on average). Given this group is only marginal in size (1.1% in the main data, 1.3% in the European Labor Force Survey), it is unlikely that these migrants bias the main results. To test this, I exclude them from the sample and re-estimate Eq 4 (Column 4 in S8 Table). The results remain substantively unchanged. As an additional robustness check of the importance of employment duration, I consider distinct age groups and interact them with the migrant indicator. If employment durations with the same employer were systematically shorter for migrants, and if this difference would drive layoff probability, we would expect heterogeneity across age groups (e.g., young native workers cannot accumulate as many additional years compared to same-age migrant co-workers). The results in Column 5 indicate that this is not the case. In fact, older migrant workers are relatively less likely (albeit statistically insignificant) to be fired compared to natives. This corroborates the assumption that the main findings are not biased by omitting employment duration in the models.

More generally, in S9 Table, I leverage two systematic tests for omitted variable bias. First, I estimate coefficient bounds under the assumption of proportional selection of observed and unobserved variables [84]. Assessing coefficient stability in combination with R-squared movements allow for the quantification of how much of the variation unobserved factors need to explain to reduce the main coefficient of interest to 0. According to the Oster-test in Panel A of S9 Table, unobserved variables (e.g., employment duration) need to be 4.2 times more informative than all observed variables to reduce the migrant coefficient to 0 (Column 4). Conversely, assuming proportionality and the proposed calculation of the hypothetical R_max_ produces exclusively positive coefficient bounds, indicating that the migrant indicator would still be associated with higher firing probability under a conservative scenario of unobserved selection.

A similar approach has been recently proposed by Cinelli and Hazlett [85], stressing that the partial R-squared of the migrant status with layoff probability can be used to infer how strong the relationship between migrant status and an unobserved confounder would need to be in order to reduce the migrant coefficient to 0. According to the estimates shown in Panel B of S8 Table, even if an unobserved confounder (or a set of confounders) would be five times more important than gender or two times more important than working part-time, migrant status would still be associated with a higher firing probability.

### 5.3. Sorting

Does firing discrimination vary across migrant groups? Because sample sizes tend to become small, I provide estimates for three disaggregates: Column 1 in S10 Table shows that citizenship is not the driving mechanism when splitting the sample into German and non-German citizens (independent of their parents’ origin). This also suggests that regulatory factors such as work permits do not play a role. As a second alternative migrant definition, I distinguish between first and second generation according to the birth country (respondent or mother/father born outside Germany). Foreign-born workers have higher firing probability with increasing shock magnitude than German-born workers (natives and second-generation migrants), as shown in Column 2. When further disaggregating the sample into native, first-, and second-generation workers in Column 3, the interaction coefficients for both migrant groups are positive and significantly different from native workers, with the effect for first-generation migrants being larger. Restricting the sample to migrants only (Column 4), however, shows that the difference between first- and second-generation migrants is not statistically significant. Together, S10 Table suggests that ethnicity–based on the parents’ origin–as opposed to citizenship is driving the differences in the firing probability.

Next, S11 Table provides two important tests for migrant sorting. Migrant sorting into industries, occupations, and contracts poses a serious identification challenge for most empirical research on discrimination trying to make aggregate statements. As elaborated above, studies have provided ample evidence for migrant sorting. While the fixed effects for industry and occupation as well as their interaction in all model specifications takes potential sorting into the former two dimensions into account, it could still be that a non-zero productivity differential to natives remains and/or that migrants sort into “bad” firms with a higher likelihood of failing under economic pressure (e.g., due to discrimination [24]). Regarding productivity differentials, I provide suggestive evidence by restricting the sample to self-employed individuals (Column 2). Estimating the model from Eq 4 (main results for comparison in Column 1) produces statistically insignificant coefficients among the self-employed. Notably, the migrant–shock interaction also has a negative sign. Regarding sorting, we do not observe company-specific characteristics in the survey. Hence, I approximate the selection of immigrants into “bad” companies by replacing the DV with an indicator of whether the respondent worked in a company that has had layoffs or in which short-time work has been introduced, independent of the respondent’s individual employment changes (Column 3; question-wording: “Have there already been layoffs in your company?” and “Has short-time work already been introduced in your company?”). In Column 4, I additionally adjust for individual layoff reported by the respondent. Neither model yields statistically significant coefficients for the migrant status indicator nor for its interaction with the magnitude of the economic shock. Consequently, the sorting of migrants does not seem large enough to systematically bias the estimated prevalence of firing discrimination.

### 5.4. Pandemic-related layoffs in perspective

**Fig 5:**
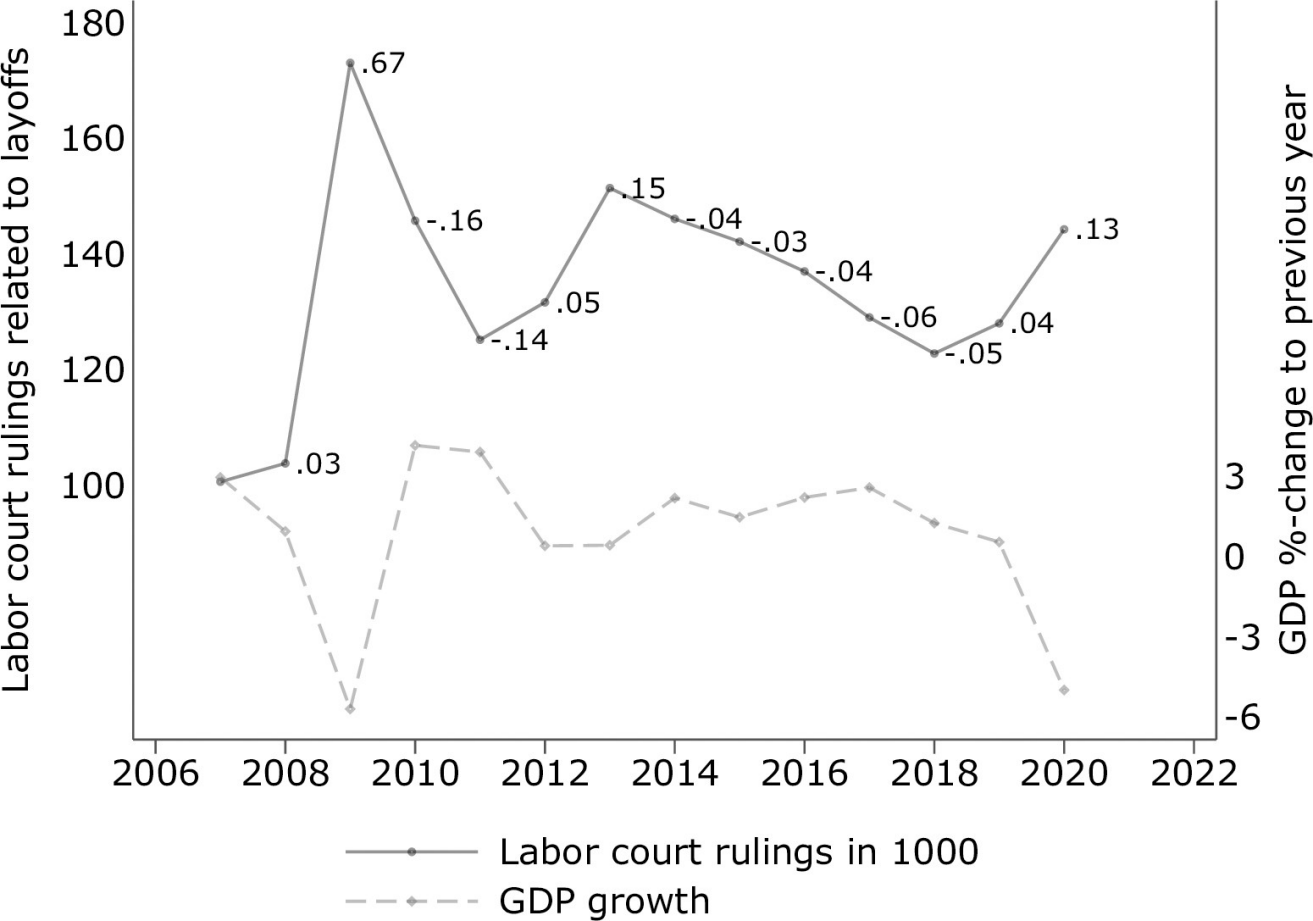
Labor court rulings and economic growth. Note: Fig presents the development of German labor court rulings dealing with layoffs over time and compares these numbers with GDP growth. Source: Federal Office of Statistics [99], own calculations.

Lastly, I present descriptive evidence on the relative importance of firing and associated discrimination during the pandemic. Fig 5 shows the development of rulings in German labor courts that dealt with appeals against layoffs. If the firing of workers (including migrants) were not to justified by objective reasons (such as seniority or child support obligations), we would expect the number of cases handled by the labor courts to increase. In fact, the percentage change in 2020 to the previous year has been substantial and is only surpassed by court decisions during the global economic crisis of 2008 onwards. Plausibly, the case number negatively correlates with economic growth, assuming that—possibly unjustified—firings occur more often during economic downturn. Considering that the economic consequences of the pandemic in Germany were not felt before April 2020, this 13% increase is even more remarkable. While Fig 5 covers any appeal against layoffs (including reasons unrelated to discrimination), it provides an indication on the importance of perceived firing discrimination in the German labor market.

## 6. Conclusion

Whether employers discriminate against ethnic and racial minorities because of their distaste against out-group members or based on quasi-rational decisions under incomplete information remains an ongoing debate in research [100, 101]. It is likely that both mechanisms are at play, and it is difficult to disentangle these mechanisms with the predominant scientific approach—that is, by measuring *hiring* discrimination.

This study assesses the heterogeneous impact of the massive and all-inclusive economic shock in the wake of the COVID-19 pandemic on migrants and natives in the German labor market. To this end, I exploit the unprecedented and sudden economic downturn, in which previously healthy firms throughout the industrial spectrum were forced to decide who to keep (and potentially put on short-time work) and who to fire, as a natural experiment. Measuring differential treatment of migrants and natives in such a situation differs from existing studies in two important respects: first, actual firing behavior is captured, whereas correspondence studies as the “gold standard” experimental approach assess invitations (for an interview) as the—less consequential—outcome (c.f. Quillian et al. [16], highlighting this potential bias). Second, and most importantly, in a firing situation, workers have gradually demonstrated their true productivity, which reduces the importance of the uncertainty-related discount factor relative to the taste-based discount factor. To further minimize the influence of statistical discrimination, I control for a number of individual, contractual, and occupational dimensions, suggesting that the remaining differences in layoff probabilities are predominantly driven by employers’ taste.

Not only are ethnic and racial minorities more at risk with regard to their health [102–104] but they are also more likely to carry the economic burden. Here, I argue that discrimination exacerbates the economic impact of the COVID-19 pandemic by showing that employers are substantially more likely to fire their workers with a migration background and to invest efforts to retain their native workforce. To the best of my knowledge, this is the first study linking job loss during the economic downturn to firing discrimination by employers.

The study setting does not come without limitations: first, the survey was administered only in the German language, which may explain the underrepresentation of migrants—apart from the fact that migrants constitute a generally hard-to-reach population. On the one hand, a more homogeneous sample in terms of language skills adds additional robustness to the findings with regard to German-speaking migrants. Because language ability is a key human capital component and main determinant for migrants’ labor market participation [76, 105], it is therefore an important element of individual productivity, too, especially in occupations that involve teamwork or customer contact (which is not captured in this questionnaire). A sample with substantial differences in the respondents’ language skills would therefore make the distinction between objective, productivity-related firing grounds and discrimination more difficult. Still, it is important to stress that the results shown here are not representative for non-German-speaking migrant workers, and hence should be regarded as a lower-bound estimate of the total extent of firing discrimination in the German labor market. Second, Adams-Prassl et al. [92] argue that the overall economic impact of the COVID-19 pandemic is less severe in Germany than in the UK or the U.S. because Germany has a well-established short-time work scheme. This would suggest that the pattern in Germany is also a lower-bound estimate of firing discrimination in general, as the analysis has shown that the migrant disadvantage increases with the severity of the economic shock. The extent of firing discrimination should thus be assessed in other contexts as well, in which industries may not have recourse to short-time work and a generous welfare state. Third, future research should investigate firing discrimination using appropriate longitudinal data. Ideally, one would adjust for unobserved factors using individual fixed effects of a representative sample that has been surveyed prior and during the pandemic. Analyzing such data that capture the specificities of layoffs and short-time work during an economic crisis and also includes detailed information on industries and occupations of individuals would certainly advance the literature on firing discrimination.

Overall, this study provides empirical evidence that discrimination continues throughout the professional stages and ends with migrants being disproportionally more likely to experience job loss than their native co-workers. This confirms the assumption that an economic downturn adds another layer of disadvantage, putting additional pressure on the immigrant workforce in times of crisis. It is thus imperative to provide rescue funds and to support firms in the hardest-hit industries of the economy—not only to enter the path of economic upturn but also to avoid a further widening of the migrant–native employment gap and the consolidation of societal cleavages along minority markers, which may have lasting adverse consequences beyond the end of the pandemic.

## Supporting information

S1 Fig Raw difference, April/May 2020 sample. (PDF)

S2 Fig Monthly excess layoffs across industries and time. (PDF)

S1 Table Sampling of online respondents, Germany, April–December 2020. (PDF)

S2 Table Summary statistics. (PDF)

S3 Table Origins of the migrant sample. (PDF)

S4 Table Industry-specific monthly changes in unemployment in Germany 2020 compared to 2019. (PDF)

S5 Table Non-linear effects corresponding to Fig 4. (PDF)

S6 Table Re-estimating Eq 4 using alternative model specifications. (PDF)

S7 Table Sub-sample and sensitivity analyses for the effect of the economic shock on layoffs. (PDF)

S8 Table Sensitivity analyses for unobserved heterogeneity. (PDF)

S9 Table Sensitivity analyses for unobserved heterogeneity. (PDF)

S10 Table Main effect under alternative migrant definitions. (PDF)

S11 Table COVID-19 response and sorting. (PDF)
