# Supplementary material for: Firing discrimination: Selective labor market responses of firms during the COVID-19 economic crisis
Source: PLoS One. 2022 Jan 31;17(1):e0262337. doi: 10.1371/journal.pone.0262337 (PMC8803145; doi:10.1371/journal.pone.0262337)
Supplement: S1 Fig — (PDF) [file pone.0262337.s001.pdf]

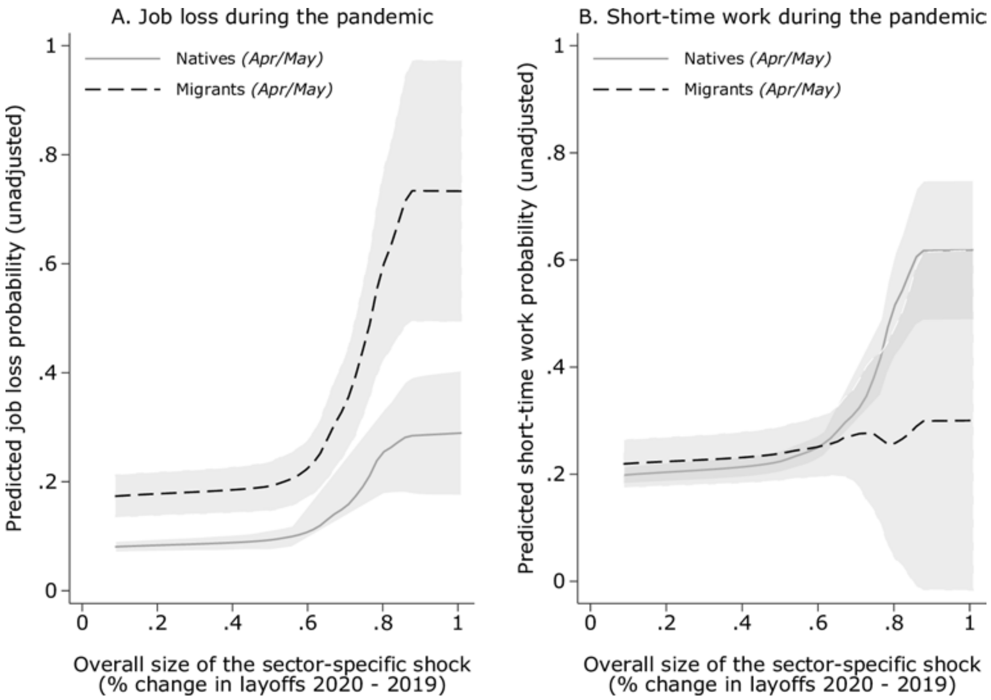

Fig S.1: Raw difference, April/May 2020 sample

Note: Fig compares unadjusted layoff and short-time work propensity as reported by survey respondents with a migration background and without by the magnitude of the industry-specific economic shock (measured by the difference in newly unemployed between 2020 and the reference month in 2019). Sample restricted to respondents surveyed in April/May 2020. Local polynomial regression (bw=0.2). Source: Federal Employment Agency [3], own calculations.
