# Supplementary material for: Firing discrimination: Selective labor market responses of firms during the COVID-19 economic crisis
Source: PLoS One. 2022 Jan 31;17(1):e0262337. doi: 10.1371/journal.pone.0262337 (PMC8803145; doi:10.1371/journal.pone.0262337)
Supplement: S2 Fig — (PDF) [file pone.0262337.s002.pdf]

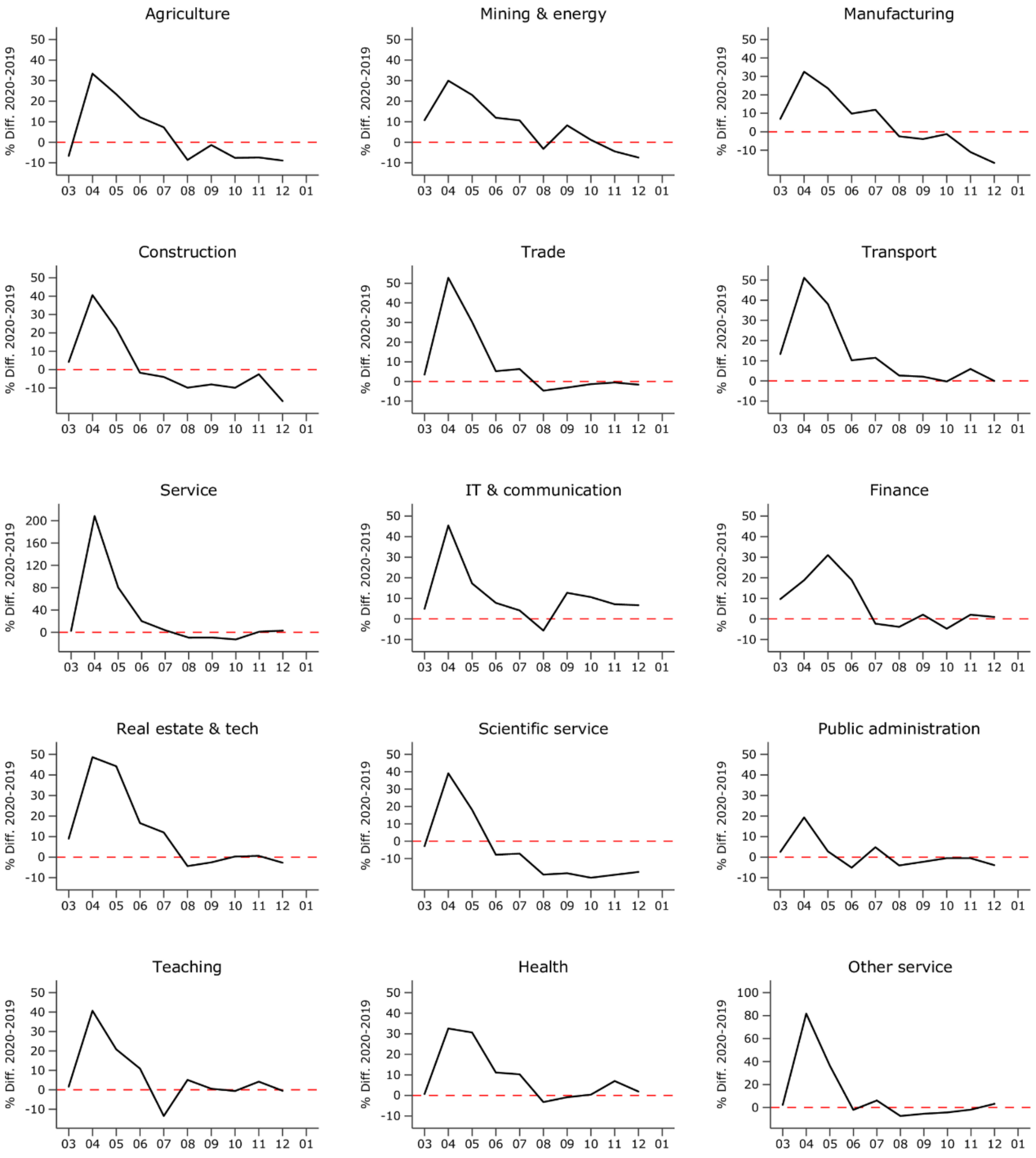

Fig S.2: Monthly excess layoffs across industries and time

Note: Fig reports the monthly difference in layoffs between 2020 and 2019 in percent as reported by the official statistics. Industry definition following Federal Office of Statistics (2021a), Source: Federal Employment Agency [3], own calculations.
