# Supplementary material for: Firing discrimination: Selective labor market responses of firms during the COVID-19 economic crisis
Source: PLoS One. 2022 Jan 31;17(1):e0262337. doi: 10.1371/journal.pone.0262337 (PMC8803145; doi:10.1371/journal.pone.0262337)
Supplement: S1 Table — (PDF) [file pone.0262337.s003.pdf]

Table S.1: Sampling of online respondents, Germany, April – December 2020

	Target share	Wave	Sampling period (start – end)	
Male	0.50	1	17.04.2020	21.04.2020
Female	0.50	2	23.04.2020	29.04.2020
		3	30.04.2020	06.05.2020
Age 18-29	0.20	4	06.05.2020	12.05.2020
30-39	0.18	5	13.05.2020	20.05.2020
40-49	0.19	6	22.05.2020	26.05.2020
50-59	0.24	7	28.05.2020	05.06.2020
60-69	0.18	8	10.06.2020	21.06.2020
		9	25.06.2020	05.07.2020
Low educational attainment	0.30	10	09.07.2020	15.07.2020
Middle	0.34	11	21.07.2020	30.07.2020
High	0.36	12	06.08.2020	17.08.2020
		13	20.08.2020	02.09.2020
		14	30.10.2020	12.11.2020
		15	13.11.2020	06.12.2020
		16	30.11.2020	16.12.2020
		17	10.12.2020	29.12.2020

Notes: Sampling shares have been targeted in every wave (sampling period). Respondents were recruited by the survey institution with the following pools (sample shares in parentheses): affiliate partner institutions (0.83), self-registration (0.14), recommendation and social media marketing (0.03). The demographic characteristics for the target shares were derived from the 2019 annual report of the Society for Integrated Communication Research (GIK) based on approximately 30,000 respondents.
