# Supplementary material for: Firing discrimination: Selective labor market responses of firms during the COVID-19 economic crisis
Source: PLoS One. 2022 Jan 31;17(1):e0262337. doi: 10.1371/journal.pone.0262337 (PMC8803145; doi:10.1371/journal.pone.0262337)
Supplement: S2 Table — (PDF) [file pone.0262337.s004.pdf]

Table S.2: Summary statistics

	survey (1)	natives (2)	migrants (3)	(3)-(2) (4)	pop. (5)
Migration background	0.162	0.000	1.000	1.000	0.260
	(0.003)	(.)	(.)	(.)	
Shock	0.174	0.171	0.191	0.019	
	(0.002)	(0.002)	(0.005)	(0.005)	
Layoff	0.120	0.071	0.131	0.060	
	(0.003)	(0.004)	(0.012)	(0.010)	
Short-time work	0.198	0.225	0.284	0.060	
	(0.004)	(0.006)	(0.016)	(0.016)	
Female	0.502	0.491	0.546	0.055	0.501
	(0.005)	(0.007)	(0.016)	(0.018)	
Age	45.180	44.147	40.210	-3.937	49.810
	(0.137)	(0.162)	(0.379)	(0.415)	
No prof. education	0.136	0.056	0.104	0.049	0.255
	(0.003)	(0.003)	(0.010)	(0.009)	
Professional education	0.542	0.578	0.459	-0.119	0.477
	(0.005)	(0.007)	(0.016)	(0.017)	
Technical training	0.108	0.122	0.119	-0.002	0.089
	(0.003)	(0.004)	(0.011)	(0.012)	
Bachelor	0.070	0.067	0.109	0.042	0.024
	(0.002)	(0.003)	(0.010)	(0.009)	
Master	0.133	0.164	0.192	0.029	0.144
	(0.003)	(0.005)	(0.013)	(0.013)	
PhD	0.010	0.014	0.016	0.002	0.012
	(0.001)	(0.002)	(0.004)	(0.004)	
Part-time contract (t_0)	0.258	0.253	0.277	0.024	
	(0.005)	(0.006)	(0.015)	(0.015)	
Fixed-term contract (t_0)	0.108	0.081	0.152	0.070	
	(0.004)	(0.004)	(0.013)	(0.011)	
Feeling overqualified (t_0)	6.326	6.315	6.492	0.177	
	(0.021)	(0.024)	(0.064)	(0.063)	
HH income (log, t_0)	9.984	9.984	10.011	0.027	
	(0.001)	(0.002)	(0.004)	(0.004)	
Military	0.003	0.004	0.004	0.001	0.001
	(0.001)	(0.001)	(0.002)	(0.002)	
Manager	0.014	0.014	0.013	-0.002	0.049
	(0.001)	(0.002)	(0.004)	(0.004)	
Academic	0.194	0.187	0.222	0.035	0.175
	(0.005)	(0.005)	(0.014)	(0.014)	
Technician	0.222	0.229	0.210	-0.018	0.192
	(0.005)	(0.006)	(0.013)	(0.015)	
Clerk	0.202	0.200	0.214	0.014	0.136
	(0.005)	(0.005)	(0.013)	(0.014)	
Vendor	0.182	0.181	0.183	0.002	0.154
	(0.005)	(0.005)	(0.013)	(0.014)	
Agriculture prof.	0.006	0.007	0.004	-0.002	0.015
	(0.001)	(0.001)	(0.002)	(0.003)	
Manual worker	0.069	0.073	0.048	-0.025	0.126
	(0.003)	(0.004)	(0.007)	(0.009)	
Assembly worker	0.050	0.050	0.045	-0.006	0.066
	(0.003)	(0.003)	(0.007)	(0.008)	
Laborer	0.057	0.056	0.057	0.002	0.081
	(0.003)	(0.003)	(0.008)	(0.008)	
Observations	11440	5292	941	6233	71.7m
Share employed	0.545				0.600

Notes: Table presents summary statistics (standard errors in parentheses) of the surveyed population (1), the employed sample without migration background (natives, 2) and the employed sample with a migration background (3). Column (4) provides differences between migrants and natives by regressing the outcome on the migrant dummy and (5) lists the German adult (>14 years) population shares for reference. t0 indicates the job-related situation as of February 2020 (i.e., prior to the spread of the pandemic). Source: Federal Employment Agency [3], own calculations.
