# Supplementary material for: Firing discrimination: Selective labor market responses of firms during the COVID-19 economic crisis
Source: PLoS One. 2022 Jan 31;17(1):e0262337. doi: 10.1371/journal.pone.0262337 (PMC8803145; doi:10.1371/journal.pone.0262337)
Supplement: S3 Table — (PDF) [file pone.0262337.s005.pdf]

Table S.3: Origins of the migrant sample

A. Migration background

	Born abroad		Non-German
	Mother/father	Respondent	citizenship
Origin	0.098	0.061	0.039

B. Most frequent countries of origin

	Observations	Share	Cumulative
	(1)	(2)	(3)
Poland	257	0.140	0.140
Turkey	174	0.095	0.236
Italy	111	0.065	0.296
Russian Federation	95	0.052	0.348
Austria	64	0.051	0.400
Romania	85	0.046	0.446
Czech Republic	78	0.043	0.489
Kazakhstan	70	0.038	0.527
Hungary	48	0.026	0.553
Croatia	44	0.024	0.577
Ukraine	41	0.022	0.600
The Netherlands	39	0.021	0.621
United States	36	0.020	0.640
France	33	0.018	0.659
Afghanistan	31	0.017	0.675
United Kingdom	28	0.015	0.691
Spain	25	0.014	0.704
Vietnam	25	0.014	0.718
Bosnia and Herzegovina	23	0.013	0.731
Greece	23	0.013	0.743
Serbia	23	0.013	0.756
Morocco	18	0.010	0.766
Portugal	16	0.009	0.774
Switzerland	16	0.009	0.783
Syria	16	0.009	0.792
Lithuania	15	0.008	0.800
India	14	0.008	0.808
Brazil	12	0.007	0.814
Iran	12	0.007	0.821
Pakistan	12	0.007	0.827
Denmark	11	0.006	0.833
The Philippines	11	0.006	0.839
Belgium	10	0.006	0.845
Kyrgyzstan	10	0.006	0.850
Slovenia	10	0.006	0.856

Notes: Table presents the type of migration background (mother/father or respondent born abroad, Panel A) and the most frequent origin countries (N>10) among the migrant sample.
