# Supplementary material for: Firing discrimination: Selective labor market responses of firms during the COVID-19 economic crisis
Source: PLoS One. 2022 Jan 31;17(1):e0262337. doi: 10.1371/journal.pone.0262337 (PMC8803145; doi:10.1371/journal.pone.0262337)
Supplement: S4 Table — (PDF) [file pone.0262337.s006.pdf]

Table S.4: Industry -specific monthly changes in unemployment in Germany 2020 compared to 2019

	2020									
	Mar	Apr	May	Jun	Jul	Aug	Sep	Oct	Nov	Dec
Agriculture	-6.6	33.4	23.4	12.2	7.3	-8.6	-1.3	-7.6	-7.4	-8.9
Mining & energy	10.8	30.0	23.0	11.9	10.7	-3.2	8.3	1.2	-4.4	-7.4
Manufacturing	7.0	32.5	23.5	9.8	11.9	-2.5	-4.0	-1.3	-11.0	-16.9
Construction	4.1	40.6	22.4	-1.7	-4.0	-9.8	-8.1	-9.9	-2.5	-17.2
Trade	3.5	52.8	30.3	5.3	6.3	-4.6	-3.1	-1.3	-0.5	-1.6
Transport	13.4	51.1	38.1	10.3	11.5	2.7	2.1	-0.3	5.9	0.0
Service	2.7	208.2	80.5	20.2	3.8	-9.4	-9.2	-12.7	1.2	3.1
IT & communication	4.9	45.4	17.3	7.8	4.1	-5.7	12.7	10.6	7.1	6.7
Finance	9.6	18.8	31.0	18.9	-2.3	-3.9	2.0	-4.8	2.0	0.9
Real estate & tech. service	9.0	48.6	44.2	16.6	12.0	-4.4	-2.5	0.3	0.6	-2.7
Scientific service	-2.8	39.1	18.1	-7.8	-7.2	-19.2	-18.4	-21.0	-19.3	-17.7
Public administration	2.5	19.3	2.8	-5.1	4.8	-4.0	-2.3	-0.5	-0.5	-3.9
Teaching	1.6	40.7	20.8	10.9	-13.5	5.1	0.5	-0.6	4.2	-0.5
Health	0.7	32.6	30.6	11.1	10.3	-3.2	-0.9	0.4	7.0	1.9
Other service	2.3	81.7	36.5	-2.0	6.1	-7.4	-5.4	-4.3	-1.9	3.2

Notes: Table presents industry-specific monthly changes in percent in newly registered unemployed individuals in 2020 compared to 2019.
Source: Federal Employment Agency [3], own calculations.
