# Supplementary material for: Firing discrimination: Selective labor market responses of firms during the COVID-19 economic crisis
Source: PLoS One. 2022 Jan 31;17(1):e0262337. doi: 10.1371/journal.pone.0262337 (PMC8803145; doi:10.1371/journal.pone.0262337)
Supplement: S5 Table — (PDF) [file pone.0262337.s007.pdf]

Table S.5: Non-linear effects corresponding to Fig 4

	Layoff	Short-time work
	(1)	(2)
Migrant	0.008	0.078
	(0.045)	(0.073)
Excess unemployment 10-19%	0.031	-0.012
Reference category: <0-9%	(0.021)	(0.032)
Excess unemployment 20-29%	0.047	-0.008
	(0.033)	(0.037)
Excess unemployment 30-39%	0.046	0.015
	(0.034)	(0.052)
Excess unemployment 40-49%	0.040	0.103*
	(0.033)	(0.056)
Excess unemployment 50+%	0.029	-0.102*
	(0.042)	(0.057)
Migrant × 10-19%	0.030	-0.034
Reference: × <0-9%	(0.045)	(0.080)
Migrant × 20-29%	0.003	-0.065
	(0.046)	(0.073)
Migrant × 30-39%	0.057	-0.044
	(0.060)	(0.085)
Migrant × 40-49%	0.098**	-0.239**
	(0.043)	(0.087)
Migrant × 50+%	0.246***	-0.140*
	(0.046)	(0.073)
Female	0.011	0.007
	(0.008)	(0.011)
Age	-0.011**	0.002
	(0.004)	(0.003)
Age2	0.000**	-0.000
	(0.000)	(0.000)
No. of children	0.009**	0.004
	(0.004)	(0.008)
Household size	-0.009	-0.009
	(0.008)	(0.016)
No formal education	0.001	0.007
Ref. = Professional educ.	(0.013)	(0.026)
Technical educ.	-0.004	-0.000
	(0.015)	(0.014)
Bachelor	0.028	-0.036
	(0.018)	(0.031)
Master	-0.007	-0.033
	(0.012)	(0.026)
PhD	0.055	-0.026
	(0.047)	(0.073)
Part-time contract	0.022	-0.012
	(0.013)	(0.020)
Fixed-term contract	0.113***	0.015
	(0.016)	(0.028)
Feeling overqualified	-0.001	0.002
	(0.002)	(0.004)
HH income (log)	-0.057	0.075
	(0.060)	(0.085)
Constant	0.757	-0.711
	(0.619)	(0.862)
R2	0.102	0.137
Observations	5473	5473
Federal state FE	16	16
Month FE	11	11
Industry FE	15	15
ISCO FE	10	10
Industry× ISCO FE	150	150

Notes: Table presents the non-linear effect of migrant status on the probability to be laid off (Model 1) and to be sent on short-time work (Model 2) using six categories of shock magnitude (% of excess unemployment) based on Equation 5. Heteroskedasticity and serial correlation robust standard errors clustered at industry level in parentheses. * p< 0.10 ** p< 0.05 *** p< 0.01. Source: Federal Employment Agency [3], own calculations.
