# Supplementary material for: Firing discrimination: Selective labor market responses of firms during the COVID-19 economic crisis
Source: PLoS One. 2022 Jan 31;17(1):e0262337. doi: 10.1371/journal.pone.0262337 (PMC8803145; doi:10.1371/journal.pone.0262337)
Supplement: S6 Table — (PDF) [file pone.0262337.s008.pdf]

Table S.6: Re-estimating Equation 4 using alternative model specifications

	PS weighted		Lagged		Monthly		COVID	No ret.
	(1)	(2)	(3)	(4)	(5)	(6)	(7)	(8)
Migrant	0.006	0.081**	0.001	0.048*	0.027	0.047	-0.017	-0.036*
	(0.026)	(0.032)	(0.030)	(0.027)	(0.016)	(0.027)	(0.026)	(0.020)
Shock	-0.006	0.098	-0.138***	-0.017	0.043**	0.011	-0.089***	0.065*
	(0.151)	(0.160)	(0.037)	(0.083)	(0.020)	(0.032)	(0.020)	(0.035)
Migrant × shock	0.250**	-0.216	0.211*	-0.114**	0.088*	-0.142*	0.212*	0.283***
	(0.124)	(0.137)	(0.107)	(0.049)	(0.047)	(0.071)	(0.106)	(0.082)
Female	0.009	0.006	0.011	0.007	0.011	0.007	-0.001	-0.004
	(0.013)	(0.016)	(0.008)	(0.011)	(0.008)	(0.011)	(0.007)	(0.006)
Age	-0.012***	0.001	-0.011**	0.002	-0.011**	0.002	-0.007**	-0.008***
	(0.004)	(0.005)	(0.004)	(0.003)	(0.004)	(0.003)	(0.003)	(0.002)
Age2	0.000***	-0.000	0.000**	-0.000	0.000**	-0.000	0.000*	0.000***
	(0.000)	(0.000)	(0.000)	(0.000)	(0.000)	(0.000)	(0.000)	(0.000)
No. of children	0.003	0.013*	0.009**	0.004	0.009**	0.004	0.007**	0.010*
	(0.005)	(0.007)	(0.004)	(0.008)	(0.004)	(0.008)	(0.002)	(0.005)
Household size	-0.007	-0.010	-0.010	-0.010	-0.009	-0.011	-0.010*	-0.005
	(0.012)	(0.016)	(0.008)	(0.016)	(0.008)	(0.016)	(0.006)	(0.008)
No formal education	-0.008	-0.019	-0.001	0.006	0.001	0.005	0.005	-0.007
Ref. = Professional educ.	(0.026)	(0.031)	(0.012)	(0.025)	(0.013)	(0.025)	(0.014)	(0.015)
Technical educ.	0.007	0.002	-0.003	-0.001	-0.003	-0.001	0.010	-0.006
	(0.015)	(0.023)	(0.015)	(0.014)	(0.015)	(0.013)	(0.012)	(0.010)
Bachelor	0.043*	-0.070***	0.029	-0.035	0.029	-0.036	0.026*	0.013
	(0.024)	(0.027)	(0.018)	(0.029)	(0.018)	(0.029)	(0.015)	(0.012)
Master	0.001	-0.040*	-0.007	-0.033	-0.006	-0.034	0.010	-0.012
	(0.019)	(0.024)	(0.012)	(0.025)	(0.012)	(0.026)	(0.009)	(0.010)
PhD	0.104	-0.043	0.054	-0.027	0.053	-0.027	0.060*	-0.047***
	(0.066)	(0.067)	(0.047)	(0.073)	(0.046)	(0.073)	(0.032)	(0.015)
Part-time contract	0.041***	-0.020	0.022	-0.013	0.021	-0.012	0.024*	0.025
	(0.015)	(0.017)	(0.013)	(0.019)	(0.012)	(0.020)	(0.014)	(0.015)
Fixed-term contract	0.110***	0.028	0.112***	0.016	0.113***	0.014	0.086***	0.068***
	(0.024)	(0.025)	(0.016)	(0.027)	(0.017)	(0.027)	(0.018)	(0.010)
Feeling overqualified	-0.001	0.003	-0.001	0.002	-0.001	0.003	-0.001	-0.001
	(0.003)	(0.004)	(0.002)	(0.004)	(0.002)	(0.004)	(0.002)	(0.002)
HH income (log)	-0.097	0.086	-0.053	0.079	-0.054	0.078	-0.045	-0.018
	(0.067)	(0.086)	(0.060)	(0.082)	(0.061)	(0.083)	(0.044)	(0.038)
Constant	1.193*	-0.811	0.757	-0.768	0.745	-0.749	0.604	0.306
	(0.667)	(0.867)	(0.623)	(0.842)	(0.623)	(0.857)	(0.442)	(0.386)
R2	0.134	0.163	0.101	0.135	0.100	0.136	0.109	0.090
Observations	5473	5473	5473	5473	5473	5473	5297	5248

Notes: Table presents the effect of migrant status on the probability to be laid off (Models 1,3,5,7,8) and to be sent on short-time work (Models 2,4,6). In Models 1,2 the sample is reweighted using post-stratification weights to closer reflect the German resident population. In Models 3,4 the shock IV is lagged (i.e., restricted to 1 month prior to the survey month). Models 5,6 estimate Equation 4 using the monthly raw changes in excess unemployment (instead of the cumulative changes). Model 7 restricts layoffs to those that reportedly were a direct consequence of the COVID-19 pandemic according to respondents, and Model 7 estimates the main model but restricts layoffs to those respondents who haven't found a new job since. Heteroskedasticity and serial correlation robust standard errors clustered at industry level in parentheses. * p< 0.10 ** p< 0.05 *** p< 0.01. Source: Federal Employment Agency [3], own calculations.
