# Supplementary material for: Firing discrimination: Selective labor market responses of firms during the COVID-19 economic crisis
Source: PLoS One. 2022 Jan 31;17(1):e0262337. doi: 10.1371/journal.pone.0262337 (PMC8803145; doi:10.1371/journal.pone.0262337)
Supplement: S7 Table — (PDF) [file pone.0262337.s009.pdf]

Table S.7: Sub-sample and sensitivity analyses for the effect of the economic shock on layoffs

	Restricted period		Time	No industry-month	
	Apr/May	Apr/Aug	invariant	heterogeneity	
	(1)	(2)	(3)	(4)	(5)
Migrant	-0.036*	-0.045**	0.009	0.038**	0.061***
	(0.018)	(0.020)	(0.024)	(0.014)	(0.014)
Shock	0.109	0.508	0.316		
	(0.073)	(0.482)	(0.238)		
Migrant × shock	0.359***	0.399***	0.392*		
	(0.056)	(0.051)	(0.209)		
Female	0.013	0.004	0.006	0.011	0.004
	(0.011)	(0.017)	(0.008)	(0.008)	(0.008)
Age	-0.010***	-0.007**	-0.012***	-0.011**	-0.020***
	(0.003)	(0.003)	(0.004)	(0.004)	(0.004)
Age2	0.000**	0.000*	0.000**	0.000**	0.000***
	(0.000)	(0.000)	(0.000)	(0.000)	(0.000)
No. of children	0.009*	0.004	0.010**	0.009**	0.010**
	(0.004)	(0.006)	(0.004)	(0.004)	(0.005)
Household size	-0.013	-0.015	-0.012	-0.010	-0.017*
	(0.008)	(0.012)	(0.008)	(0.008)	(0.008)
No formal education	-0.020	0.000	0.006	0.000	0.024
Ref. = Professional educ.	(0.023)	(0.045)	(0.013)	(0.013)	(0.022)
Technical educ.	0.015	0.006	-0.005	-0.003	0.001
	(0.018)	(0.015)	(0.016)	(0.015)	(0.019)
Bachelor	0.037*	0.003	0.033*	0.029	0.027
	(0.018)	(0.031)	(0.017)	(0.018)	(0.017)
Master	-0.008	-0.032	-0.004	-0.007	0.008
	(0.014)	(0.026)	(0.011)	(0.012)	(0.015)
PhD	0.029	0.009	0.044	0.053	0.056
	(0.034)	(0.042)	(0.046)	(0.046)	(0.048)
Part-time contract	0.022	0.041*	0.026*	0.022	0.033**
	(0.013)	(0.022)	(0.013)	(0.013)	(0.014)
Fixed-term contract	0.083***	0.060*	0.111***	0.112***	0.164***
	(0.019)	(0.033)	(0.016)	(0.016)	(0.018)
Feeling overqualified	-0.004	-0.006	-0.001	-0.001	0.002
	(0.004)	(0.004)	(0.002)	(0.002)	(0.002)
HH income (log)	-0.088**	-0.014	-0.056	-0.056	-0.020
	(0.037)	(0.074)	(0.056)	(0.060)	(0.062)
Constant	1.087**	0.264	0.805	0.777	0.534
	(0.398)	(0.739)	(0.566)	(0.619)	(0.639)
R2	0.114	0.135	0.064	0.099	0.122
Observations	3680	2022	5473	5473	6199
Federal state FE	16	16	16	16	16
Month FE	2	5	11	11	11
Sector FE	15	15		15	15
ISCO FE	10	10	10	10	10
Sector × ISCO FE	150	150		150	150

Notes: Models 1-3 estimate Equation 4, with Model 1 (2) restricting the observation period to respondents surveyed between April and May (August). Model 3 replaces the cumulative monthly shock measure with a time-invariant indicator capturing the cumulative excess layoffs at the industry level between Mar and Dec 2020 (rel. to 2019) for all respondents (independent of the interview date). Models 4, 5 show the shock-invariant extent of firing discrimination during 2020. Model 5 additionally incorporates respondents who indicated employment as of March 1, 2020 but for whom no industry and/or occupation could be identified, grouped in separated categories of industry and ISCO. Heteroskedasticity and serial correlation robust standard errors clustered at industry level in parentheses. * p< 0.10 ** p< 0.05 *** p< 0.01. Source: Federal Employment Agency [3], own calculations.
