# Supplementary material for: Firing discrimination: Selective labor market responses of firms during the COVID-19 economic crisis
Source: PLoS One. 2022 Jan 31;17(1):e0262337. doi: 10.1371/journal.pone.0262337 (PMC8803145; doi:10.1371/journal.pone.0262337)
Supplement: S8 Table — (PDF) [file pone.0262337.s010.pdf]

Table S.8: Sensitivity analyses for unobserved heterogeneity

	Employment duration in the European Labor Force Survey			Main survey	
	Country of birth	Citizen-ship	Interaction	No DE-born foreign cit.	Age groups
	(1)	(2)	(3)	(4)	(5)
German-born	-1.849				
	(1.523)				
German citizen		0.931***			
		(0.074)			
German-born foreign citizen			-0.836***		
Ref. = German born German citizen			(0.113)		
Foreign-born German citizen			1.901		
			(1.528)		
Foreign-born foreign citizen			0.921		
			(1.531)		
Migrant				-0.009	0.128**
				(0.025)	(0.053)
Shock				-0.025	0.013
				(0.031)	(0.035)
Migrant × shock				0.265***	
				(0.082)	
Age 26-34					-0.071**
Ref = age < 26					(0.028)
Age 36-45					-0.095**
					(0.032)
Age 46-55					-0.101***
					(0.034)
Age 56-65					-0.105***
					(0.031)
Migrant × Age 26-34					-0.086
					(0.051)
Migrant × Age 36-45					-0.100
					(0.062)
Migrant × Age 46-55					-0.106*
					(0.056)
Migrant × Age 56-65					-0.103
					(0.060)
R2	0.368	0.369	0.369	0.102	0.103
Observations	250,849	250,849	250,849	5415	5473
Individual controls	yes	yes	yes	yes	yes
Residence FE	23	23	23		
ISCO FE	10	10	10	10	10
Federal state FE				16	16
Month FE				11	11
Industry FE				15	15
Industry× ISCO FE				150	150

Notes: Models 1-3 estimate the employment duration in the same company using European Labor Force Survey data. Model 1 defines migrant status based on country of birth (Germany vs. other) and Model 2 based on citizenship (German vs. other). Model 3 show the interaction of citizenship and country of birth. Model 4 re-estimates the main Equation 4, excluding respondents born in Germany but without Germany citizenship. Model 5 defines five age groups and interacts them with migrant status (full sample). All models adjusted for the same confounders as in Table 1 (except HH income (log), feeling overqualified, and part-time contract in Model 1-3). Fixed effects in Models 1-3: education (9 categories), residence duration (23 year categories), and occupation (10 categories) fixed effects. * p< 0.10 ** p< 0.05 *** p< 0.01. Source: Federal Employment Agency [3], Eurostat [83], own calculations.
