# Supplementary material for: Firing discrimination: Selective labor market responses of firms during the COVID-19 economic crisis
Source: PLoS One. 2022 Jan 31;17(1):e0262337. doi: 10.1371/journal.pone.0262337 (PMC8803145; doi:10.1371/journal.pone.0262337)
Supplement: S9 Table — (PDF) [file pone.0262337.s011.pdf]

Table S.9: Sensitivity analyses for unobserved heterogeneity

Panel A. Oster test for unobservable selection and coefficient stability

	Baseline	Controlled	Identified set	δ for τ=0
	(1)	(2)	(3)	(4)
Migrant *(Layoffs)*	0.060	0.038	[0.003;0.038]	4.287
	(0.018)	(0.014)		
Migrant *(Short-time work)*	0.059	0.028	[0.009;0.028]	2.886
	(0.025)	(0.024)		

Panel B. Cinelli and Hazlett sensitivity analysis

	Controlled	$R2_{Y\sim D\|X}$	$RV_{q=1}$	$RV_{q=1,p=0.05}$
Layoffs	(1)	(2)	(3)	(4)
Migrant	0.038	0.25%	4.84%	2.25%
	(0.014)			
		$R2_{Y\sim Z\|X,D}$	$R2_{D\sim Z\|X}$	
Bounds		(5)	(6)	
Z = 1x female		0.03%	0.00%	
Z = 5x female		0.17%	0.02%	
Z = 1x part-time contract		0.11%	0.01%	
Z = 2x part-time contract		0.21%	0.02%	

Notes: *Panel A* presents results of the Oster test for unobservable selection and coefficient stability. Column 1 shows the uncontrolled effect of migrant status on layoff and short-time work probability, respectively; Column 2 the main model's controlled effect (without shock interaction). The identified set's upper bound is set by the controlled effect; its lower bound is based on δ=1 and R_{max} = 1.3R = 0,129 (0.175 for short-time work). Column for shows the estimated δ that would reduce the migrant coefficient to 0. *Panel B* show the results of the Cinelli and Hazlett sensitivity analysis. Column 1 shows the main coefficient for migrant on layoff probability (without shock interaction). Column 2 shows how much of the residual outcome migrant status explains after adjusting for observables. The Robustness Value in Column 3 (and in Column 4 after accounting for sampling uncertainty) shows how much confounders need to explain of the residual variation of both, layoffs and migrant status to explain away the effect. The bounds in Columns 5 and 6 compare the strength of unobserved confounders with an observed one (female, working part-time). Robust standard errors in parentheses. * p< 0.10 ** p< 0.05 *** p< 0.01. Source: Federal Employment Agency [3], own calculations.
