# Supplementary material for: Firing discrimination: Selective labor market responses of firms during the COVID-19 economic crisis
Source: PLoS One. 2022 Jan 31;17(1):e0262337. doi: 10.1371/journal.pone.0262337 (PMC8803145; doi:10.1371/journal.pone.0262337)
Supplement: S10 Table — (PDF) [file pone.0262337.s012.pdf]

Table S.10: Main effect under alternative migrant definitions

	Citizen-ship	Country of birth	Citizen/CoB	
			Interaction	No natives
	(1)	(2)	(3)	(4)
German citizen	-0.002			
	(0.024)			
Foreign born		-0.013		
		(0.020)		
German born migrant			-0.014	
			(0.038)	
Foreign born migrant			-0.010	0.018
			(0.022)	(0.034)
Shock	0.001	0.000	-0.024	0.559**
	(0.035)	(0.032)	(0.035)	(0.258)
German citizen × shock	-0.046			
	(0.060)			
Foreign born × shock		0.123*		
		(0.065)		
German born migrant × shock			0.361**	
			(0.147)	
Foreign born migrant × shock			0.134*	-0.138
			(0.068)	(0.168)
Female	0.009	0.011	0.011	-0.006
	(0.008)	(0.008)	(0.008)	(0.024)
Age	-0.012**	-0.011**	-0.011**	-0.019*
	(0.004)	(0.004)	(0.004)	(0.010)
Age2	0.000**	0.000**	0.000**	0.000
	(0.000)	(0.000)	(0.000)	(0.000)
No. of children	0.009**	0.009**	0.009**	0.007
	(0.004)	(0.004)	(0.004)	(0.012)
Household size	-0.009	-0.009	-0.009	0.018
	(0.008)	(0.008)	(0.008)	(0.035)
No formal education	0.009	0.007	-0.000	-0.028
Ref. = Prof. educ.	(0.013)	(0.012)	(0.012)	(0.044)
Technical educ.	-0.001	-0.000	-0.004	0.065
	(0.016)	(0.016)	(0.015)	(0.041)
Bachelor	0.034*	0.032*	0.030	0.042
	(0.018)	(0.018)	(0.018)	(0.069)
Master	-0.005	-0.004	-0.006	0.012
	(0.012)	(0.012)	(0.012)	(0.038)
PhD	0.062	0.057	0.055	0.066
	(0.049)	(0.046)	(0.046)	(0.125)
Part-time contract	0.021	0.021	0.022	0.038
	(0.013)	(0.013)	(0.013)	(0.057)
Fixed-term contract	0.113***	0.114***	0.114***	0.046
	(0.016)	(0.016)	(0.016)	(0.029)
Feeling overqualified	-0.001	-0.001	-0.001	-0.011
	(0.002)	(0.002)	(0.002)	(0.007)
HH income (log)	-0.055	-0.056	-0.052	-0.233
	(0.061)	(0.061)	(0.061)	(0.153)
Constant	0.793	0.790	0.732	2.932*
	(0.632)	(0.625)	(0.631)	(1.626)
R2	0.096	0.097	0.102	0.237
Observations	5481	5519	5473	770
Federal state FE	16	16	16	16
Month FE	11	11	11	11
Industry FE	15	15	15	15
ISCO FE	10	10	10	10
Industry× ISCO FE	150	150	150	150

Notes: Table presents layoff probabilities for alternative classifications of migrant status. Model 1 defines migration status as 1 if a person does not hold German citizenship and 0 otherwise. Model 2 defines migration status as 1 if the person was born abroad. Model 3 defines migration status as 0 if a respondent and both parents were born in Germany, as 1 if a person was born in Germany but not her parents (2nd generation), and as 2 if the respondent was born outside Germany (1st generation). Model 4 restricts the sample to migrants (at least one parent born abroad) only. Heteroskedasticity and serial correlation robust standard errors clustered at industry level in parentheses. * p< 0.10 ** p< 0.05 *** p< 0.01. Source: Federal Employment Agency [3], own calculations.
