# Supplementary material for: Firing discrimination: Selective labor market responses of firms during the COVID-19 economic crisis
Source: PLoS One. 2022 Jan 31;17(1):e0262337. doi: 10.1371/journal.pone.0262337 (PMC8803145; doi:10.1371/journal.pone.0262337)
Supplement: S11 Table — (PDF) [file pone.0262337.s013.pdf]

Table S.11: COVID-19 response and sorting

	Main model	Productivity and sorting		
		Self-empl.	Firm response	
	(1)	(2)	(3)	(4)
Migrant	-0.008	0.112	0.042	0.044
	(0.026)	(0.102)	(0.031)	(0.027)
Shock	-0.022	0.363	-0.126*	-0.121*
	(0.033)	(0.384)	(0.060)	(0.062)
Migrant × shock	0.243***	-0.302	0.132	0.075
	(0.081)	(0.285)	(0.115)	(0.100)
Individual layoff				0.233***
				(0.013)
Female	0.011	-0.011	-0.012	-0.014
	(0.008)	(0.047)	(0.014)	(0.013)
Age	-0.011**	-0.015	-0.005	-0.003
	(0.004)	(0.013)	(0.004)	(0.004)
Age2	0.000**	0.000	0.000	0.000
	(0.000)	(0.000)	(0.000)	(0.000)
No. of children	0.009**		0.014**	0.012*
	(0.004)		(0.006)	(0.006)
Household size	-0.009		-0.006	-0.004
	(0.008)		(0.012)	(0.011)
No formal education	0.000	0.040	0.044**	0.044*
Ref. = Prof. educ.	(0.013)	(0.056)	(0.020)	(0.022)
Technical educ.	-0.004	-0.005	0.036	0.037*
	(0.015)	(0.074)	(0.021)	(0.018)
Bachelor	0.029	0.060	0.022	0.016
	(0.018)	(0.066)	(0.019)	(0.019)
Master	-0.007	-0.013	0.004	0.005
	(0.012)	(0.059)	(0.012)	(0.011)
PhD	0.054	-0.053	-0.035	-0.048
	(0.046)	(0.099)	(0.036)	(0.029)
Part-time contract	0.022	0.173***	-0.014	-0.019**
	(0.013)	(0.046)	(0.008)	(0.008)
Fixed-term contract	0.113***		0.076***	0.049**
	(0.016)		(0.019)	(0.018)
Feeling overqualified	-0.001		-0.001	-0.001
	(0.002)		(0.004)	(0.004)
HH income (log)	-0.054	-0.081	0.037	0.049
	(0.060)	(0.207)	(0.059)	(0.059)
Constant	0.760	0.264	-0.598	-0.776
	(0.625)	(2.066)	(0.653)	(0.671)
R2	0.101	0.253	0.091	0.120
Observations	5473	492	5468	5468
Federal state FE	16	16	16	16
Month FE	11	11	11	11
Sector FE	15	15	15	15
ISCO FE	10	10	10	10
Sector × ISCO FE	150	150	150	150

Notes: Table provides suggestive evidence for equal migrant-native productivity and the absence of systematic sorting into firms that were disproportionately affected by the pan-demic and compares the coefficients to the main Model 1 as in Table 1. Model 2 restricts the sample to respondents who have been self-employed as of March 2020, suggesting that self-employed migrants do not differ from natives in terms of job loss. Models 3,4 replace DV individual layoffs with a binary indicator whether layoffs or short-time work have been introduced at the firm level as reported by the respondent. Model 4 additionally adjusts for reported individual layoff. Heteroskedasticity and serial correlation robust standard errors clustered at industry level in parentheses. * p< 0.10 ** p< 0.05 *** p< 0.01. Source: Federal Employment Agency [3], own calculations.
